# Experimental and DFT Studies of Au Deposition Over WO_3_/g-C_3_N_4_ Z-Scheme Heterojunction

**DOI:** 10.1007/s40820-019-0345-2

**Published:** 2019-12-19

**Authors:** Muhammad Humayun, Habib Ullah, Junhao Cao, Wenbo Pi, Yang Yuan, Sher Ali, Asif Ali Tahir, Pang Yue, Abbas Khan, Zhiping Zheng, Qiuyun Fu, Wei Luo

**Affiliations:** 1 Engineering Research Center for Functional Ceramics of the Ministry of Education, School of Optical and Electronic Information, Huazhong University of Science and Technology, Wuhan, 430074 People’s Republic of China; 2 China-EU Institute for Clean and Renewable Energy, Huazhong University of Science and Technology, Wuhan, 430074 People’s Republic of China; 3 Environment and Sustainability Institute (ESI), University of Exeter, Penryn Campus, Penryn, Cornwall TR10 9FE UK; 4 College of Engineering and Applied Sciences, Nanjing University, Nanjing, 210093 People’s Republic of China; 5 Department of Chemistry, Abdul Wali Khan University, Mardan, Khyber Pakhtunkhwa 23200 Pakistan

**Keywords:** Polymeric g-C_3_N_4_, Plasmonic Au, Charge separation, Solar H_2_ production, DFT calculations

## Abstract

A typical Z-scheme system is composed of two photocatalysts which generate two sets of charge carriers and split water into H_2_ and O_2_ at different locations. Scientists are struggling to enhance the efficiencies of these systems by maximizing their light absorption, engineering more stable redox couples, and discovering new O_2_ and H_2_ evolutions co-catalysts. In this work, Au decorated WO_3_/g-C_3_N_4_ Z-scheme nanocomposites are fabricated via wet-chemical and photo-deposition methods. The nanocomposites are utilized in photocatalysis for H_2_ production and 2,4-dichlorophenol (2,4-DCP) degradation. It is investigated that the optimized 4Au/6% WO_3_/CN nanocomposite is highly efficient for production of 69.9 and 307.3 µmol h^−1^ g^−1^ H_2_ gas, respectively, under visible-light (*λ* > 420 nm) and UV–visible illumination. Further, the fabricated 4Au/6% WO_3_/CN nanocomposite is significant (i.e., 100% degradation in 2 h) for 2,4-DCP degradation under visible light and highly stable in photocatalysis. A significant 4.17% quantum efficiency is recorded for H_2_ production at wavelength 420 nm. This enhanced performance is attributed to the improved charge separation and the surface plasmon resonance effect of Au nanoparticles. Solid-state density functional theory simulations are performed to countercheck and validate our experimental data. Positive surface formation energy, high charge transfer, and strong non-bonding interaction via electrostatic forces confirm the stability of 4Au/6% WO_3_/CN interface.

**Electronic supplementary material:**

The online version of this article (10.1007/s40820-019-0345-2) contains supplementary material, which is available to authorized users.

## Highlights

- Experimental and density functional theory studies were performed for Au decorated WO_3_/g-C_3_N_4_ Z-scheme heterojunction.
- The amount optimized 4Au/6WO_3_/CN composite exhibited high performance for H_2_ evolution and 2,4-DCP degradation due to the improved charge separation in WO_3_/g-C_3_N_4_ composite and the surface plasmon resonance ct of Au.

**Electronic supplementary material:**

The online version of this article (10.1007/s40820-019-0345-2) contains supplementary material, which is available to authorized users.

## Graphical abstract

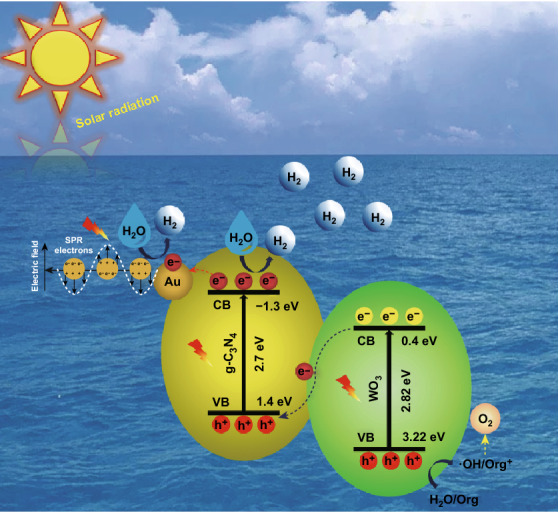

## Introduction

The emerging energy and environmental issues as a result of the rapid consumption of fossil fuels became a hot topic of current researchers. Scientists across the globe are searching for low cost, renewable, clean and green form of energy, which could replace the traditional fossil fuels [1]. In recent decades, H_2_ has been recognized as a precious and essential element of the de-carbonized sustainable fuel system. Hydrogen could play a vital role in low-carbon future, facilitating a more clean, low cost, and pollution free energy. Hence, counter-balancing the electricity as a zero-carbon energy carrier for storage and transportation, and reducing global CO_2_ emission to two-thirds [2]. Semiconductor photocatalysis is one of the promising techniques that can be used for clean H_2_ production (renewable green fuel) and elimination of environmentally persistent pollutants [3, 4].

Since the first report on photocatalysis over TiO_2_ semiconductor in 1972 [5], great efforts have been devoted to design and develop semiconductor photocatalysts that can efficiently harvest solar energy and convert it into chemical fuels. TiO_2_ is the mostly investigated semiconductor photocatalyst owing to its proper conduction and valence band levels for redox reactions [6]. Nevertheless, the wide band gap (3.2 eV) seriously limits its photocatalytic performance because of the insufficient solar light absorption (i.e., ca. 4%). Since, the solar spectrum comprises the main part as visible-light region (i.e., 46%) [7]. Accordingly, for efficient photocatalysis, the fabrication of visible-light responsive photocatalysts is of great significance.

In past few years, narrow band gap inorganic semiconductors (visible-light responsive), based on transition or post-transition metal-oxides with *d*^0^, *d*^10^, *f*^0^ configurations [8–11], (oxy)nitrides [12, 13], and sulfides [14, 15] have been investigated. However, these semiconductors exhibited low photocatalytic activity because of high charge recombination rate and small surface area.

Beside inorganic semiconductors, an organic metal-free polymeric carbon nitride (g-C_3_N_4_) semiconductor received worldwide scientific attention owing to its high performance for solar fuel generation under visible-light irradiation, as investigated by Wang et al. [16]. With characteristic band gap of 2.7 eV, g-C_3_N_4_ exhibit band edges set over water redox potentials. The conduction band minimum of g-C_3_N_4_ is located at − 1.3 V (pH = 7), while its valence band maximum is located at 1.4 V. Further, g-C_3_N_4_ is only composed of earth abundant C and N elements. Due to the presence of aromatic C–N heterocycles, g-C_3_N_4_ is thermally stable up to 600 °C in air condition. In addition, due to the presence of strong van der Waals forces between the layered structures of g-C_3_N_4_, it is chemically stable in most of the solvents. Thus, g-C_3_N_4_ is thermodynamically suitable and stable for water reduction and oxidation reactions [17]. Yet, the performance of g-C_3_N_4_ in photocatalysis is poor because of the inadequate visible-light response, fast charge carriers recombination rate, and small surface area of bulk material [18]. To overcome these challenging drawbacks and to promote the performance of g-C_3_N_4_ in photocatalysis, various morphological and compositional modification strategies have been proposed [19–25]. The most important strategy is to promote charge carriers separation in g-C_3_N_4_, by constructing type-II and Z-scheme heterojunctions in combination with other semiconductors [26, 27]. Recently, Jiang et al. [28] published their work on the synthesis of TiO_2_/g-C_3_N_4_ type-II heterojunctions that showed obvious performance for H_2_ evolution and pollutants degradation in comparison to the single g-C_3_N_4_. In another report by Acharya et al. [29], the Pt activated LaFeO_3_/g-C_3_N_4_ Z-scheme heterojunctions exhibited remarkably enhanced photoactivity for H_2_ generation compared to the bare g-C_3_N_4_. This was attributed to the promoted charge carriers separation in the resultant Z-scheme heterojunction. Notably, the synthesis of Z-scheme heterojunction has become an effectual strategy not only to boost up the separation efficiency of induced charge carriers at the interface junction but also to conserve excellent redox ability [30, 31].

In addition to the heterojunction formation, the deposition of noble metals like gold (Au), silver (Ag), and platinum (Pt) over g-C_3_N_4_ exhibited significantly improved photocatalytic performance. The reason might be due to the avoidance of charge recombination in the space-charge vicinity, collectively with the extended light absorption because of the surface plasmon resonance (SPR) effect [32–34]. The SPR effect of noble metal nanoparticles produces an intense local electromagnetic field which speeds up the rate of formation of electrons and holes in g-C_3_N_4_. Furthermore, the favorable Fermi energy level of these noble metal nanoparticles make possible the separation of photo-induced charge carriers, which in turn enhances the quantum efficiency of g-C_3_N_4_. Further, the electrons transfer shifts the Fermi energy level to a more negative potential, thereby enhancing the reducibility of electrons in the Fermi energy level close to the conduction band of g-C_3_N_4_ [35]. According to Li et al. [36] sulfur-doped g-C_3_N_4_/Au/CdS Z-scheme heterojunctions exhibited remarkably improved visible-light catalytic activities. In our previous report [37], 2% Au loaded SnO_2_/g-C_3_N_4_ composite exhibited significantly improved visible-light catalytic activity for H_2_ evolution. Thus, it is much meaningful to fabricate g-C_3_N_4_-based Z-scheme heterojunction and further to utilize the SPR effect of Au for efficient photocatalysis. Although, several works have been reported on the fabrication of WO_3_/g-C_3_N_4_ Z-scheme heterojunctions [38, 39]. In fact, no attention has been focused toward the photo-deposition of Au on WO_3_/g-C_3_N_4_ Z-scheme system and the detail investigations based on the experimental and theoretical studies.

Herein, we have fabricated Au decorated WO_3_/g-C_3_N_4_ Z-scheme heterojunctions. The amount optimized sample showed significantly improved catalytic activities under visible and UV–visible irradiation. It is confirmed by various experiments that the enhanced activities of g-C_3_N_4_ are attributed to the drastically improved charge carriers separation and transfer via the coupled nano-sized WO_3_ and further to the promoted charge carriers separation and redox ability via the SPR effect of decorated Au nanoparticles. In addition, periodic density functional theory (DFT) simulations have been accomplished in parallel to experiments, to validate and countercheck the experimental results. The simulated surface formation energy confirms the stability of Au/WO_3_/g-C_3_N_4_ interface junction and consequences non-bonding interaction. This work will provide detail knowledge on the understanding of photocatalytic mechanisms and fabrication of g-C_3_N_4_-based high performance visible-light catalysts for solar H_2_ production and pollutants degradation.

## Experimental Section

The reagents used in this work were of analytical grade. De-ionized (DI) water was used in all the experiments.

### Fabrication of g-C_3_N_4_

To fabricate g-C_3_N_4_, 10 g of dicyandiamide precursor was taken in a semi-covered ceramic crucible and kept in muffle furnace. The precursor was annealed in air at 550 °C (temp-ramp = 5 °C min^−1^) for 2 h. After self-cooling to room temperature, the product was re-calcined at 550 °C for 2 h to obtain g-C_3_N_4_ with sheet-like morphology. Finally, the g-C_3_N_4_ with yellow color was crushed into fine powder and used in various experiments and characterizations.

### Fabrication of WO_3_

The WO_3_ nanoparticles were fabricated via hydrothermal method. Approximately 0.7 g of Na_2_WO_4_·2H_2_O precursor was dissolved into 70 mL DI water and then 10 mL of HCl (35%) was drop wise added to it. The solution was hydrothermally treated in a 100 mL Teflon-lined autoclave at 160 °C for 6 h. After cooling down, the sample was collected through centrifugation by washing with ethanol and DI water. The product was kept in oven to dry overnight at 65 °C and then annealed at 350 °C (temp-ramp = 5 °C min^−1^) for 2 h.

### Fabrication of WO_3_/g-C_3_N_4_ HeteroJunctions

The WO_3_/g-C_3_N_4_ heterojunctions were prepared via wet-chemical method. Typically, for each composite, 2 g of g-C_3_N_4_ (base material) was dispersed into 40 mL of water–ethanol mixture (1:1). Then, different mass ratio percentage of WO_3_ (i.e., 2, 4, 6, and 8%) was added to each sample. The samples were kept under stirring for 4 h and then dried in oven at 65 °C overnight. The powder samples were calcined in muffle furnace at 450 °C (temp-ramp = 5 °C min^−1^) for 2 h. Finally, the composites samples were represented by *x*WO_3_/CN, where x stands for percentage composition of WO_3_.

### Fabrication of Au/WO_3_/g-C_3_N_4_ Heterojunctions

The Au/WO_3_/CN heterojunctions were prepared by photo-deposition method. Different mass ratios (i.e., 1, 2, 3, 4, and 5%) of Au nanoparticles were deposited on the surface of amount optimized 6WO_3_/CN composite. For each sample, 1 g of the composite powder was dispersed in 80 mL methanol contained in a 250 mL-volume round bottom flask. Then, the required amount of Au solution (HAuCl_4_·4H_2_O) prepared in DI water was added to it. The flask was properly covered and the mixture was bubbled with N_2_ gas for 30 min to remove the dissolved O_2_ and to create an inert atmosphere for photo-reduction of Au nanoparticles. Each sample was kept under magnetic stirring and irradiated under UV-light (*λ* = 200–400 nm) with a 300 W Xe-lamp for 2 h. Then, the samples were collected by centrifugation, meanwhile washed with DI water. Finally, the samples were dried in oven at 65 °C. The dark color samples were labeled as *y*Au/6WO_3_/CN, where y stands for different mass ratio of Au to the optimized 6WO_3_/CN photocatalyst.

### Materials Characterization

The X-ray diffraction (XRD) patterns of the catalysts were analyzed by 08 X’Pert3 powder X-ray diffractometer (PANalytical, Netherlands). The UV–visible absorption spectra were recorded with Lambda-35-UV/Vis spectrophotometer (PerkinElmer, USA), and BaSO_4_ was used a reference for samples calibration. The morphology of the photocatalysts was investigated by a German scanning electron microscope (SEM, Geminisem, 300–7112) and transmission electron microscope (FTEM, Talos F200x, FEI, Netherlands). The elemental mappings images and elemental analysis was performed with scanning electron microscope (Geminisem, 300–7112). The elemental chemical analysis of the samples was carried out with an AXIS-ULTRA DLD-600 W, X-ray photoelectron spectrometer (XPS, Kratos, Japan). The functional groups and surface composition of the samples were investigated by a 23 VERTEX-70 Fourier transform infrared (FT-IR) spectrometer (Bruker, Germany), using KBr disk as a sample diluent. The surface photovoltage spectra (SPV) were obtained with a home-built device, set with lock-in amplifier (SR830, made in USA) and light chopper (SR540, made in USA). The photoluminescence (PL) spectroscopy analysis was performed with a 22 FP-6500 fluorescence spectrometer (Jasco, Japan), at excitation wavelength of 325 nm. The thermo-gravimetric analysis (TGA) was performed with the help of TGA8000 (Perkin Elmer, USA) in the range of 30–800 °C, under air condition. The Brunauer–Emmett–Teller (BET) specific surface area and pore size distribution of the optimized samples were measured by N_2_ adsorption–desorption technique (ASAP-2020, USA). The PEC I-V curves measurement was carried out with a three electrode system configuration containing Ag/AgCl as the reference electrode, a Pt foil as the counter electrode and sample film as the working electrode. The electrolyte was 0.5 M L^−1^ Na_2_SO_4_ aqueous solution. The electron paramagnetic resonance (EPR) spectroscopy was performed with an EPR spectrometer (JEOL-FA200, made in Japan) at room temperature, and the 5,5-Dimethyl-1-pyrroline-*N*-oxide (DMPO) was used as a trapping reagent.

### Hydroxyl Radical (^·^OH) Measurement

The ^·^OH amount generated by each photocatalyst was measured by coumarin fluorescence method. About 0.05 g of each photocatalyst was dispersed into 40 mL of the coumarin aqueous solution (0.001 M) contained in a 100 mL volume glass reactor. To achieve adsorption equilibrium, the solution was stirred in dark for 30 min and then irradiated under visible light (420 nm cutoff) for 1 h with a 300 W Xe-lamp. After that the desired amount of solution was centrifuged and subjected for analysis of 7-hydroxycoumarin through fluorescence spectrometer (Jasco, Japan) at excitation wavelength of 390 nm.

### Photoactivities Measurements

The photoactivities of samples were evaluated for H_2_ generation from H_2_O and CH_3_OH mixture, and for degradation of 2,4-dichlorophenol pollutant. The tests for H_2_ generation were performed in an online H_2_ generation system (PerfectLight, Beijing). For each experiment, 0.1 g of photocatalyst was dispersed in DI water/methanol (80 mL/20 mL) mixture contained in a glass reactor. Prior to the reaction, the mixture was kept under magnetic stirring and evacuated for 1 h to remove the dissolved O_2_ and CO_2_ in water. After that the samples were irradiated under Visible (420 nm cutoff) and UV–visible light with a 300 W Xe-lamp. Amount of H_2_ evolved was detected by an inline gas chromatograph (7920-TCD, N_2_ gas carrier, CEAULIGHT). The stability of the amount optimized photocatalyst for H_2_ evolution was explored by four photocatalytic recyclable tests (each cycle of 4 h).

The 2,4-DCP degradation tests were carried out in a 100 mL quartz reactor. For each experiment, 0.2 g of catalyst was dispersed in 80 mL of 10 mg L^−1^ 2,4-DCP solution. The samples were kept in dark under magnetic stirring for 30 min to attain the adsorption equilibrium and then irradiated under visible light for 2 h using a 300 W Xe-lamp. The desired amount of solution was centrifuged and taken into a quartz cell for measurement of the 2,4-DCP concentration. The concentration of 2,4-DCP was analyzed with a Lambda-35 Perkin-Elmer spectrophotometer (made in USA) at the characteristic absorption wavelength of 2,4-DCP (i.e., 285 nm). The degradation percentage (*η*%) of 2,4-DCP was calculated by using Eq. 1:

1 $$\eta {\text{\% }} = \frac{{C_{0} - C}}{{C_{0} }} \times 100\%$$

where *C*_0_ is the initial concentration of pure 2,4-DCP, *C* is the concentration at time *t*, and *η*% is the degradation rate of 2,4-DCP.

### Computational Methodology

DFT simulations were completed with Quantum-ATK [40] and results are interpreted on VESTA and Virtual NanoLab Version 2019.3.3 [41]. WO_3_ with Hall symmetry space group of P2_1/c [42, 43] is used as such. The unit cell of WO_3_ is stable at room temperature which contains 8 W, 24 O atoms, and 8 oxygen corner-sharing octahedrons. After optimization of the bulk unit cell lattice parameters, a super-cell (2 × 2 × 2) was build, from which WO_3_(001) slab was constructed. The slab thickness was equal to 8 conventional unit cells of WO_3_ which has thickness of 15 Å with 256 atoms. This thickness was enough to make sure that the slab center can represent the bulk phase of WO_2_. It is widely reported that the [001] termination exhibit low surface formation energy and therefore represent the most stable phase of WO_3_ [44]. The stability of various slabs is established from their positive surface formation energy (~ 0.38 J m^−2^) and electrostatic potential. A single layer of CN is considered for simulations, where three gold atoms are dispersed on the surface of Au/CN to generate 4% Au decorated CN. H_2_ atoms are employed to passivate the systems, especially for the monolayers of CN, Au/CN and their corresponding heterojunctions. The generalized gradient approximation (GGA) with Perdew–Burke–Ernzerhof (PBE) exchange correlation function and double-zeta polarized (DZP) basis-set is used for the crystal structure and energy optimization because of its superior over hybrid pseudo-potentials [45]. In this work, a linear combination of atomic orbitals (LCAO) technique is used for W, Au, C, N, H, and O atoms [46]. A 5 × 5 × 5 Monkhorst–Pack k-grid and energy-cutoff of 1200 eV is used for WO_3_ unit cell, while a 5 × 5 × 1 k-point-mesh is used for WO_3_ slabs. Thus, 7 × 7 × 7 Monkhorst–Pack k-grid and energy-cutoff of 900 eV is used for monolayers of CN, WO_3_/CN, and Au/CN, and 5 × 5 × 1 k-point-mesh with 1200 eV cutoff-energy for 6WO_3_/CN and 4Au/6WO_3_/CN heterostructures. We tried many methods for band-structure simulations such as GGA/PBE, meta-GGA, SGGA, and GGA + U. Due to ferromagnetic nature (naturally magnetic behavior) of W, the GGA + U method can accurately reproduce its electronic properties, especially band structure, etc. Thus, the band-structure calculations were completed with GGA + U, where the U value is set to accurately represent the experimental data. Partial density of states (PDOS), band-structure and electrostatic potential maps are also designed. The DFT-occupied and unoccupied DOS are regarded as the VB and CB edges, respectively [47].

## Results and Discussion

### Structural Characterization and Chemical Composition

**Fig. 1:**
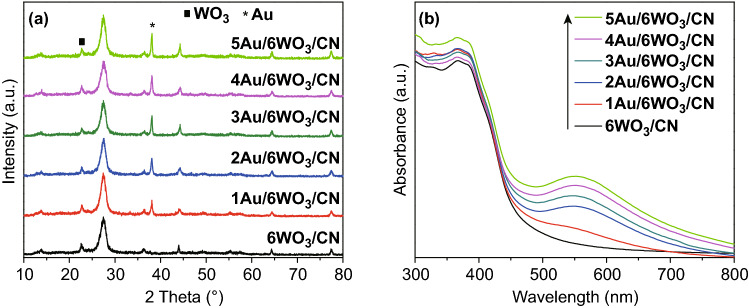
**a** XRD patterns, and **b** UV–Vis absorption spectra of 6WO_3_/CN and *y*Au/6WO_3_/CN composites

The XRD patterns of pristine CN, WO_3_, and *x*WO_3_/CN photocatalysts are provided in Fig. S1a. It is clear that CN exhibits two distinct diffraction peaks located at 2*θ* = 13.0° and 27.7°, respectively, which can be indexed to the characteristic (100) and (002) planes of g-C_3_N_4_ [48]. The strong peak at 2*θ* = 27.7° is ascribed to the inter-layer stacking interaction of conjugated rings with an inter-layer distance of 0.325 nm [49]. The XRD patterns of pure WO_3_ display a monoclinic-phase constitution with prominent diffraction peaks at 2*θ* of 23.17°, 24.40°, 33.50°, and 36.22°, indexed to the characteristic planes (002), (200), (022), and (202), respectively, (JCPDS No. 72-1465) [50]. Interestingly, it can be seen that coupling of WO_3_ does not influence the crystal structure of CN, while the characteristic (002) peak of WO_3_ appeared in the *x*WO_3_/CN composites and its intensity gradually enhanced with the increase in WO_3_ content. Further, it can be seen that the characteristic (002) peak of CN significantly decreased in the *x*WO_3_/CN composites, which may be due to the high dispersion of monoclinic WO_3_ over the surface of CN [51–53]. The XRD patterns of *y*Au/6WO_3_/CN composites are shown in Fig. 1a. It is obvious that after decorating Au, the characteristic diffraction peaks of Au at 2*θ* values of 38.2° and 44.4° can be observed in the *y*Au/6WO_3_/CN composites. These peaks are ascribed to the face-centered cubic structure of Au with (111) and (200) crystal planes, respectively [32]. These peaks intensity slightly enhanced with the increase in amount of Au.

The UV–visible absorption spectra of CN, WO_3_, and *x*WO_3_/CN photocatalysts are provided in Fig. S1b. The band gap energies of CN, WO_3_, and *x*WO_3_/CN photocatalysts were predicted from intercept of tangents to plots of (*αhν*)^1/2^ versus *hν* as displayed in Fig. S1c. As obvious, pure WO_3_ and CN photocatalysts exhibit band gap energies of 2.82 and 2.7 eV, respectively. It is worth noting that coupling of WO_3_ does not influence the band gap of CN. The absorption spectra of *y*Au/6WO_3_/CN nanocomposites are shown in Fig. 1b. In comparison to the 6WO_3_/CN nanocomposite, the absorption spectra of *y*Au/6WO_3_/CN composites exhibit an additional absorption peak occupying a wavelength range of 500–650 nm, centering at 560 nm. This absorption band corresponds to the SPR effect of Au, confirming that Au is successfully deposited on the CN surface [54]. It can be seen clearly that intensity of the SPR absorption peak greatly enhanced with the increase in amount of Au loading. Further, the estimated band gaps (Fig. S1d) reveals that deposition of Au does not influence the band gap of 6WO_3_/CN composite. In fact, the SPR absorption can induce a strong local electric field around the interface that enhances the light absorption of the surrounding molecules and accelerates the separation rate of photo-induced charges leading to the enhanced photocatalytic activities [55].

SEM images were taken to examine the morphology of CN, WO_3_, and *x*WO_3_/CN photocatalysts. Figure S2a reveals that CN exhibit stacked layers of ultra-thin flat surface nanosheets with thickness in the range of 80–100 nm. Figure S2b reveals that monoclinic WO_3_ exhibit nanoplates like morphology with thickness in the range of 30–50 nm. Figure S2c–f shows the SEM micrographs of *x*WO_3_/CN composites. The adhering of WO_3_ particles on the surface of CN nanosheets can be clearly observed. To investigate the elemental-distribution morphology of 6WO_3_/CN photocatalyst, energy-dispersive spectroscopy (EDS) elemental-mapping analysis was carried out as shown in Fig. S3a–e. The red, magenta, cyan, and green colors correspond to the distribution of C, N, W, and O elements, respectively, which further confirmed the existence of C, N, W, and O elements and their respective atomic percentage composition is shown in Fig. S3f inset. The SEM micrographs of *y*Au/6WO_3_/CN nanocomposites (Fig. S4a–e) show that Au nanoparticles are successfully deposited on the surface of 6WO_3_/CN nanocomposite. Furthermore, EDS spectrum of 4Au/6WO_3_/CN nanocomposite shows the presence of C, N, W, O, and Au elements (Fig. S4f). To explore the distribution morphology of the optimized 4Au/6WO_3_/CN nanocomposite, EDS elemental-mapping analysis was carried out as shown in Fig. S5a–f. The elements C, N, W, O, and Au are evenly distributed in the composite.

**Fig. 2:**
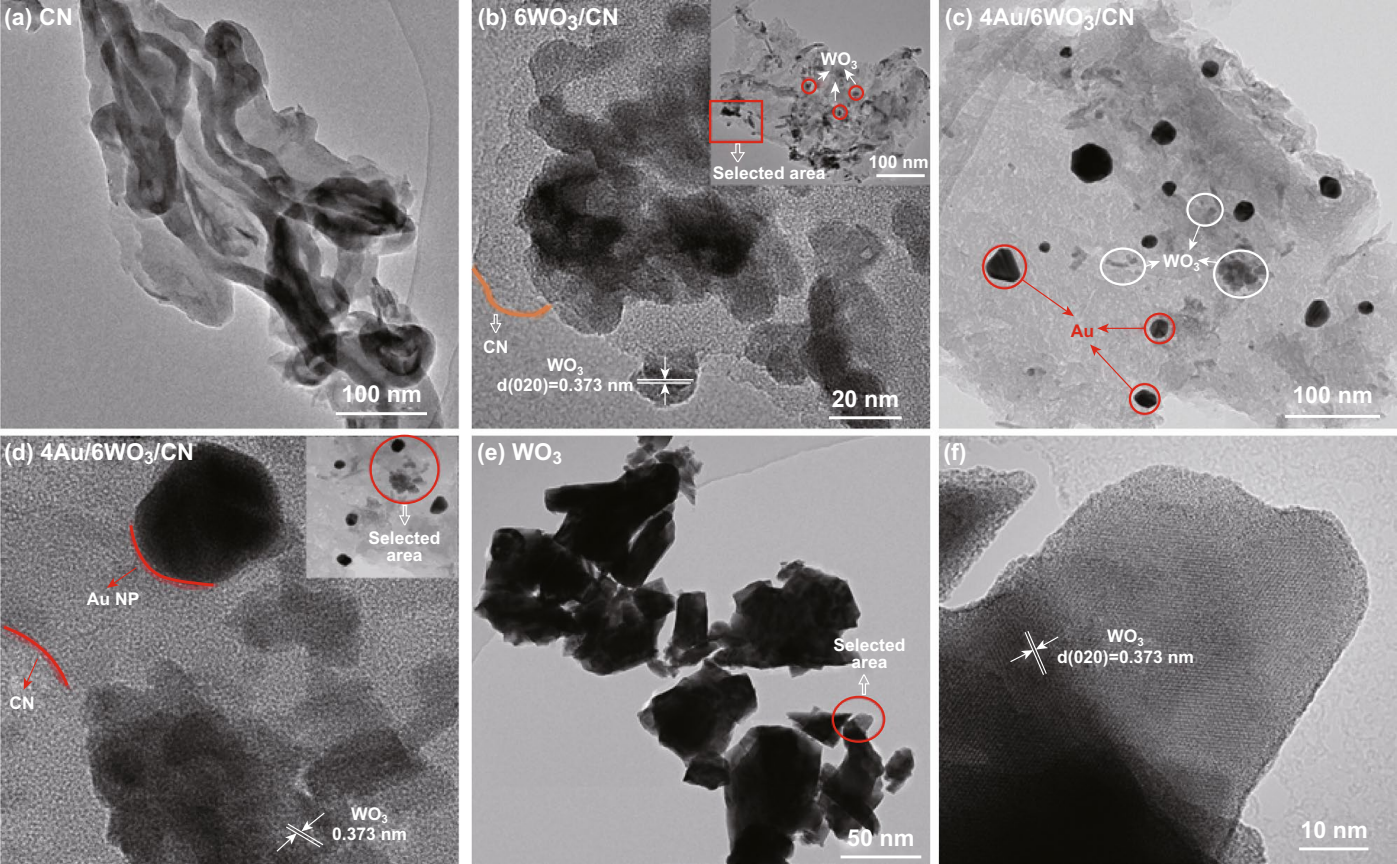
**a** TEM image of CN, **b** HRTEM image with inset TEM image of 6WO_3_/CN composite. **c** TEM image of 4Au/6WO_3_/CN composite. **d** HRTEM image of 4Au/6WO_3_/CN composite with selected area TEM image as inset. **e** TEM image of WO_3_, and **f** HRTEM image of WO_3_

TEM and high resolution TEM images of CN, 6WO_3_/CN, 4Au/6WO_3_/CN, and WO_3_ photocatalysts are shown in Fig. 2. Figure 2a clearly shows that CN exhibits nanosheet like essence. The TEM image of 6WO_3_/CN nanocomposite (Fig. 2b inset) shows that nanosize WO_3_ particles with average diameter of 50 nm are well dispersed on the CN surface. The selected area HRTEM image of 6WO_3_/CN nanocomposite (Fig. 2b) shows that the lattice-fringes with *d*-spacing of 0.373 nm correspond to the (002) plane of WO_3_. The TEM image of 4Au/6WO_3_/CN nanocomposite (Fig. 2c) clearly demonstrates that except for WO_3_, the Au nanoparticles with average diameter in the range of 20–30 nm are well decorated on the surface of CN. The selected area HRTEM image of 4Au/6WO_3_/CN nanocomposite (Fig. 2d) clearly shows that WO_3_ and Au nanoparticles are in close contact with the CN surface. The TEM image of WO_3_ is shown in Fig. 2e. As shown in the HRTEM image (Fig. 2f), the *d*-spacing of 0.373 nm corresponds to the (002) plane of WO_3_.

The BET surface area and pore volume of CN, 6WO_3_/CN, and 4Au/6WO_3_/CN photocatalysts were explored by BET N_2_ adsorption–desorption technique. As shown in Fig. S6a, the adsorption isotherm curves of all photocatalysts exhibit a type-IV behavior with H_3_ hysteresis loops, signifying mesoporous structures. The observed specific surface areas of CN, 6WO_3_/CN, and 4Au/6WO_3_/CN photocatalysts are 45.6, 51, and 54 m^2^ g^−1^, respectively. The pore diameter values of the photocatalysts were calculated by Barrett–Joyner–Halenda (BJH) models, and the plots are provided in Fig. S6b. The pore diameter distribution curves of the photocatalysts show that the average pore diameters of the photocatalysts are in the range of 2 nm. The BET results reveal that surface area of the photocatalysts may not be the key factor affecting the catalytic activities of the photocatalysts [56].

To reveal about the chemical composition of photocatalysts, FT-IR spectra were obtained. The FT-IR spectra of CN, WO_3_, and *x*WO_3_/CN photocatalysts are shown in Fig. S6C. As obvious, pure CN exhibit characteristic absorption peaks at 810, 1242, 1307, 1410, 1463, 1562, 1641, and 3200–3500 cm^−1^. The absorption peak at 810 cm^−1^ is ascribed to the out-of-plane bending modes of triazine-units. The peaks in range of 1240–1590 cm^−1^ correspond to stretching-mode of C–N heterocycles. The peaks at 1307 and 1641 cm^−1^ are assigned to the C–N and C=N stretching-modes in CN, respectively. The broad peak in the region of 3200–3500 cm^−1^ can be ascribed to the NH stretching-modes and the surface-adsorbed OH^−^ groups [57]. The pure WO_3_ shows characteristic absorption peaks in the region of 500–900 cm^−1^, which corresponds to the (O–W–O) stretching vibrations in a monoclinic-type WO_3_. The absorption peak at 1625 cm^−1^ is due to the (O–H) bending-vibration modes of the coordinated water and the broad band in the region of 3200–3500 cm^−1^ is due to the stretching-vibration mode of the surface-adsorbed OH^−^ groups [58]. In case of *x*WO_3_/CN composites, the characteristic absorption band of WO_3_ does not appear which may be due to its low content. Further, Au deposition does not influence the structural morphology of 6WO_3_/CN composite (Fig. S6d).

**Fig. 3:**
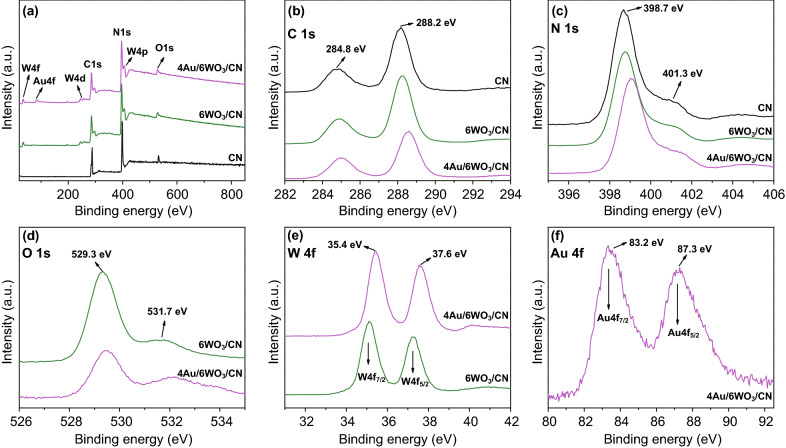
**a** XPS survey spectra, **b** high-resolution C 1*s* XPS spectra, and **c** high-resolution N 1*s* XPS spectra of CN, 6WO_3_/CN, and 4Au/6WO_3_/CN photocatalysts. High-resolution **d** O 1*s* XPS spectra, and **e** W 4*f* XPS spectra of 6WO_3_/CN and 4Au/6WO_3_/CN photocatalysts. **f** High-resolution Au 4*f* XPS spectra of 4Au/6WO_3_/CN photocatalyst

To further confirm the elemental chemical composition of photocatalysts, XPS spectra were measured as provided in Fig. 3. As clear from Fig. 3a, the XPS survey spectra of 6WO_3_/CN is composed of C, N, W, and O elements, while that of 4Au/6WO_3_/CN contains C, N, W, O, and Au elements. The high-resolution C 1*s* XPS (Fig. 3b) clearly demonstrate that CN display two prominent peaks at binding energies of 284.8 and 288.2 eV, which is ascribed to the adventitious carbon used as a reference for the sample calibration and the C–N–C coordination, respectively [51]. Worth noting that after coupling WO_3_, these binding energy peak positions are little shifted toward high energy side, and even more shifted after photo-deposition of Au nanoparticles. This may be due to the interfacial-charge transfer in the composites. The high-resolution N 1*s* XPS spectra of CN, 6WO_3_/CN, and 4Au/6WO_3_/CN are provided in Fig. 3c. The N 1*s* XPS spectrum of CN display two intense peaks located at binding energies of 398.7 and 401.3 eV, which corresponds to the *sp*^2^-hybridized N in triazine-rings (C–N=C) and tertiary (N–(C)_3_) groups, respectively [59]. It can be observed that the N 1*s* XPS peaks of 6WO_3_/CN and 4Au/6WO_3_/CN composites are little shifted toward high energy side due to the electron delocalization effect. The high-resolution O 1*s* XPS spectra of 6WO_3_/CN and 4Au/6WO_3_/CN composites (Fig. 3d) exhibit two peak at 529.3 and 531.7 eV, which correspond to the lattice oxygen of WO_3_and the hydroxyl (–OH) groups, respectively [60]. The high-resolution W 4*f* XPS spectra of 6WO_3_/CN and 4Au/6WO_3_/CN composites (Fig. 3e) show the presence of two peaks at 34.4 and 37.6 eV, which are ascribed to the W4*f*_7/2_ and W4*f*_5/2_ orbital splitting of W^6+^ in WO_3_, respectively [61]. The high-resolution Au 4*f* XPS spectrum of 4Au/6WO_3_/CN composite (Fig. 3f) displays two prominent peaks at binding energies of 83.2 and 87.3 eV, which are ascribed to the Au 4*f*_7/2_ and Au 4*f*_5/2_ orbital splitting of Au, respectively [32].

TGA results of CN, 6WO_3_/CN, and 4Au/6WO_3_/CN photocatalysts are shown in Fig. S7. Worth noting that a rapid weight loss for pure CN can be observed in the temperature range of 600–680 °C, which can be attributed to the disintegration/burning of CN. However, a rapid decrease in the weight of 6WO_3_/CN and 4Au/6WO_3_/CN composites can be observed in the temperature range of 550–680 °C. Similar to other CN-based photocatalysts [62], this decrease in weight can be attributed to the slightly decrease in thermal stability of pure CN by coupling WO_3_ and depositing Au. The content of CN in the 6WO_3_/CN and 4Au/6WO_3_/CN composites was calculated from the remaining weights after treating the photocatalysts over 700 °C, which are 92% and 90.1% for the 6WO_3_/CN and 4Au/6WO_3_/CN photocatalysts, respectively.

### First Principle Study of 4Au/6WO_3_/CN Heterojunction

In order to well understand the structure, binding, and the electronic properties of 4Au/6WO_3_/CN composite, periodic density functional theory (DFT) simulations have been carried out. The optimized relaxed geometries (models) of WO_3_, 6WO_3_/CN, and 4Au/6WO_3_/CN are shown in Fig. S8. In these heterojunctions, both CN and Au decorated CN have strong non-bonding interactions with the surface atoms of WO_3_. These models are further employed for the electronic properties simulations. The geometric parameters of the simulated relaxed crystal structure of the unit cell are compared with the already reported crystallographic parameters of WO_3_, which conclude that PBE/GGA accurately reproduces the experimental data. Moreover, the applied theoretical protocol was counterchecked from the simulated per atom cohesive formation energy of W, O, C, H, Au, and N. The calculated band gap and formation energy of WO_3_ are 2.80 eV and 0.38 J m^−2^, respectively, which has nice correlation with the already reported work [43]. All theoretical band-edge energies are simulated at vacuum phase. The details of surface formation energies simulations can be found from the previous reports [63, 64].

The density of state (DOS) and partial DOS (PDOS) plots along with band structure of WO_3_ are given in Figs. S9a and S10. It can be seen that VB and CB positions of WO_3_ are located at − 7.72 and − 4.9 eV, respectively, (vs. vacuum). The occupied 5*d* band of W is lower in energy than that of the O 2*p* band which consequence O 2*p* VB at − 7.72 eV and W 5*d* CB at − 4.9 eV with a band gap of 2.82 eV (vs. vacuum) (Fig. S10). Thus, the VB is dominated by oxygen atoms while W dominates CB of WO_3_. Moreover, the PDOS of CN along with band structure is shown in Figs. S9b and S11. In this case, the VB and CB are located at − 5.9 and − 3.2 eV, respectively, (vs. vacuum). The band edge positions of both of WO_3_ and CN are consistent with the already reported work that validates our theoretical methods.

The interaction of CN and Au/CN with that of WO_3_ surface produces some inter-bands in the band gaps of 6WO_3_/CN (Figs. S12–S15). This statement also corroborates our experimental data. This strong interaction between WO_3_ and CN and Au/CN with WO_3_, enhances the overall catalytic activity of resulted material (vide infra). The reason behind this is the strong hybridizations of the bonding and anti-bonding orbitals of W, O, C, N, H, and Au (Figs. S13–S15). At Fermi energy of 5.09 eV, the vacuum-phase VBM and CBM values of WO_3_/CN are slightly changed as that of parent WO_3_. However, the N and C of CN have produced some extra bands in the band gap of WO_3_ as can be seen from Fig. S12a, b. These extra bands (also called trap centers) can trap the electron–hole recombination which consequently enhances the photocatalytic activity.

The PDOS and DOS plots of 4Au/6WO_3_/CN are given in Fig. S16. The bonding and anti-bonding orbitals of W, O, C, N, H, and Au constitute band gap and band edge positions. Again, similar but severe behavior of Au/CN is observed in 4Au/6WO_3_/CN photocatalyst. Figure S16a, b shows that both the bonding and anti-bonding orbitals of Au/CN are prominent compared to that of the CN in 6WO_3_/CN system. In addition, the VBM and CBM of 4Au/6WO_3_/CN are moved toward more positive potentials, aligned with the reduction potential of water to efficiently reduce H_2_O and produce H_2_. The strong hybridizations and perturbation of these edges positions validate our experimentally proposed Z-scheme behavior of 4Au/6WO_3_/CN system (vide infra).

Moreover, the band alignment of WO_3_, CN, Au/CN, 6WO_3_/CN, and 4Au/6WO_3_/CN heterojunction were calculated from difference of vacuum-energy (*E*_vac_) and Fermi energy level (*E*_F_). *E*_vac_ is the energy of a stationary electron in vacuum nearby the surface. The simulated electrostatic potential maps of WO_3_, CN, Au/CN, 6WO_3_/CN, and 4Au/6WO_3_/CN heterojunction along the *Z*-direction are shown in Figs. S17–S19. The work functions of WO_3_, CN, Au/CN, 6WO_3_/CN, and 4Au/6WO_3_/CN photocatalysts are 6.30, 4.38, 4.24, 5.09, and 4.95 eV, respectively. A large difference in work functions and band-edge potentials (VBM and CBM) of CN and WO_3_ led us to predict that photo-generated electrons of CN cannot transfer thermodynamically to the CB of WO_3_ in 6WO_3_/CN heterojunction. Instead, the excited holes in the VB of CN recombine with the photo-generated electrons of WO_3_ which consequently enhance charge carriers separation and photo-reduction ability of CN.

According to our previous report [37], the work function of Au is 5.1 eV which is slightly higher than that of 6WO_3_/CN composite (5.09 eV). So, in case of 4Au/6WO_3_/CN heterojunction, the photo-generated electrons (CB) of CN will transfer to the surface of Au nanoparticles. Meanwhile, the excited holes of CN will recombine to the excited electrons of WO_3_, which significantly promote charge carriers separation and photocatalytic activity. Comparative analysis of Fig. S17C, D led us to conclude that work function of 4Au/6WO_3_/CN (4.95 eV) is lower than that of the 6WO_3_/CN (5.09 eV). So, it is inferred that Au has accepted electronic cloud density of CN (donor).

**Fig. 4:**
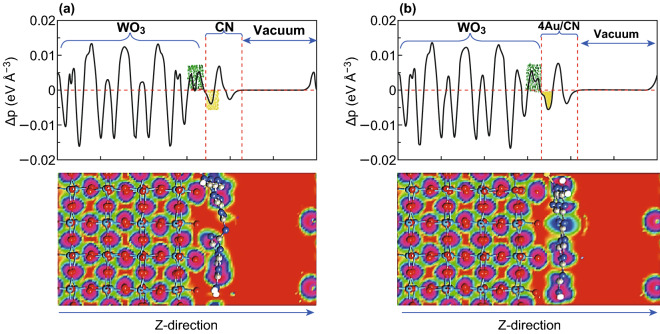
Average electron difference density along with electrostatic potential maps of **a** 6WO_3_/CN and **b** 4Au/6WO_3_/CN composites. The green and yellow shaded areas denote electron accumulation and donation, respectively. (Color figure online)

Finally, inter-charge transfer in 6WO_3_/CN and 4Au/6WO_3_/CN heterojunctions is calculated from charge-density difference (CDD), as shown in Fig. 4. The green and yellow shaded areas, respectively, represent the charge acceptance and donation. Charge analysis of these Figures reveals that charge distribution occur at the interface of both 6WO_3_/CN and 4Au/6WO_3_/CN heterojunctions. In addition, we can see that bulk region of WO_3_ has no appreciable change, especially the far side area of the interface. So, we can predict, this type of charge distribution may result a strong interaction between Au/CN and WO_3_. Analysis of charge redistribution at the interfaces of these species led us to conclude that electrons (CB) of CN and Au/CN directly reduce water while its holes recombine with the excited electrons of WO_3_. So, holes at VBM of WO_3_ oxidize H_2_O and produce O_2_ while the electrons at CN, especially, at Au/CN surface reduce water and generate H_2_. The amount of charge density difference between 6WO_3_/CN and 4Au/6WO_3_/CN heterojunctions is ~ 0.073 and 0.091 electrons, respectively, (Bader charge analysis). The higher charge transformation in 4Au/6WO_3_/CN compared to that of the 6WO_3_/CN can also be visualized from the EDD maps (green shaded areas), as shown in Fig. 4a, b. In a nutshell, this charge accumulation and donation ignite an electric field at the interface of 4Au/6WO_3_/CN heterojunction, which consequently separate electrons and holes (vide supra).

### Photo-Generated Charge Behavior

**Fig. 5:**
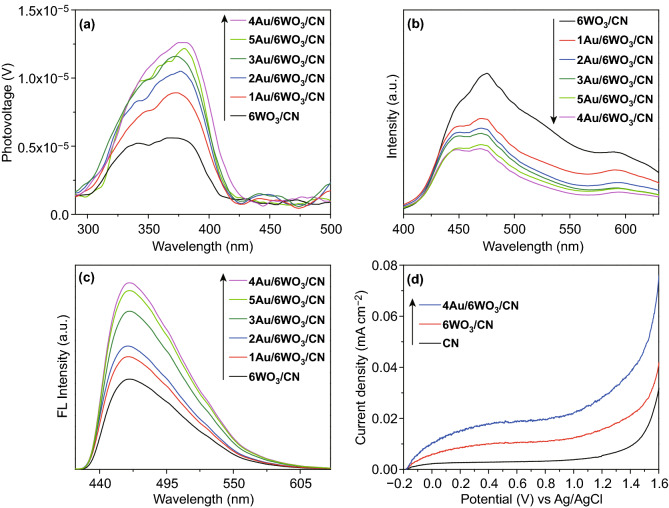
**a** Surface photovoltage (SPV) spectra, **b** photoluminescence (PL) spectra, and **c** fluorescence spectra related to the ^·^OH amount of 6WO_3_/CN and *y*Au/6WO_3_/CN composites. **d** Photoelectrochemical *I*–*V* curves of CN, 6WO_3_/CN, and 4Au/6WO_3_/CN

To reveal the photo-generated charge carrier’s properties of CN, *x*WO_3_/CN, and *y*Au/6WO_3_/CN photocatalysts, SPV and PL characterizations were carried out. The SPV is a sensitive and non-destructive technique mainly used to explore the photo-physics of photo-generated charges in the semiconductor nanomaterials. The SPV signals for nanomaterials mainly originate from photo-generated charge carriers separation via the diffusion phenomenon [65]. In fact, higher is the photo-generated charge carriers separation, stronger will be the SPV signal and vice versa. The SPV signals of CN, WO_3_, and *x*WO_3_/CN photocatalysts measured under air condition are shown in Fig. S20a. The low-intensity SPV signal of CN mainly resulted from the fast recombination rate of photo-generated charges. It is important to note that the SPV response of CN is drastically improved after coupling WO_3_ and the intense signal is detected for 6WO_3_/CN photocatalyst. Worth noting, the SPV signal intensity of 8WO_3_/CN photocatalyst is greatly reduced because of the over the excess amount of WO_3_ that covers the CN surface and acts as a charge recombination center. As shown in Fig. 5a, the SPV signal intensity of 6WO_3_/CN composite is further improved after photo-deposition of Au nanoparticles, and the highest intensity signal is detected for the amount optimized 4Au/6WO_3_/CN composite. Based on the SPV signals, it is concluded that loading proper amount of WO_3_ and Au could greatly enhance the charge carrier’s separation of CN, leading to the superior photocatalytic performance.

Further, the charge carrier’s separation is confirmed by the PL measurement. The PL is a highly sensitive technique used to investigate surface-properties of nanomaterials. Generally, it tells us about the photo-generated charge carriers trapping, migration, transfer, oxygen vacancies, active sites, and surface defects in semiconductor nanomaterials [66–68]. The PL spectra of CN, WO_3_, and *x*WO_3_/CN photocatalysts are displayed in Fig. S20b. In fact, the stronger PL signal reveals high charge carrier’s recombination rate and vice versa. Notably, CN shows strong PL signal with emission peak centering at 460 nm. This is ascribed to the band–band transition in bare CN. Thus, it is clear that charge carrier’s recombination rate in CN is quite fast. Obviously, the PL intensity of CN is drastically reduced after coupling WO_3_ and the low-intensity signal is detected for 6WO_3_/CN photocatalyst. This confirms that charge carrier’s recombination rate is greatly suppressed by the synergistic effect of the two components system. Interestingly, Fig. 5b shows that PL intensity of 6WO_3_/CN composite is further decreased after photo-deposition of Au nanoparticles and the lowest intensity signal is detected for 4Au/6WO_3_/CN composite. The PL results are in accordance with the SPV results.

As mentioned in the previous report [69], the fluorescence spectra corresponding to ^·^OH amount could effectively reveal the charge separation in photocatalysis. Thus, the fluorescence spectra of CN, WO_3_, *x*WO_3_/CN, and *y*Au/6WO_3_/CN photocatalysts were measured to further confirm the improved charge carriers separation. For this, the coumarin-fluorescent technique was used to measure the ^·^OH amount. As widely accepted, the coumarin reacts with ^·^OH and generate luminescent 7-hydroxycoumarin which has a characteristic fluorescence emission peak at about 450 nm wavelength. Figure S20c shows that CN exhibit weak fluorescence signal which reflects the negligible amount of ^·^OH generated. Further, the fluorescence intensity peak of *x*WO_3_/CN composites become stronger with an increase in percentage composition of WO_3_ especially that of the amount optimized 6WO_3_/CN composite. Interestingly, as can be seen from Fig. 5c, after photo-deposition of Au nanoparticles, the fluorescence intensity peak of 6WO_3_/CN composite becomes stronger and the highest fluorescence intensity peak is recorded for 4Au/6WO_3_/CN composite, which is in accordance to the SPV and PL results.

To further reveal the improved charge carriers separation, photoelectrochemical *I*–*V* curves analysis of CN, 6WO_3_/CN, and 4Au/6WO_3_/CN photocatalysts were performed in Na_2_SO_4_ electrolyte (0.5 M) at 0.4 V bias versus the Ag/AgCl electrode as shown in Fig. 5d. From PEC *I*–*V* curves result, it can be concluded that the photocurrent density of CN is not obvious. However, compared to the CN, an abrupt increment in the photocurrent density response is observed for 6WO_3_/CN composite. Interestingly, the photocurrent density of 4Au/6WO_3_/CN photocatalyst is much stronger signifying that charge carrier separation is drastically enhanced.

The photo-generated electron–hole pair’s separation was further investigated by the measurement of electrochemical-impedance spectra of CN, 6WO_3_/CN, and 4Au/6WO_3_/CN photocatalysts as shown in Fig. S20d. The EIS Nyquist plots arc radius of 4Au/6WO_3_/CN photocatalyst is significantly reduced compared to that of the 6WO_3_/CN composite and bare CN. These results demonstrate that the 4Au/6WO_3_/CN photocatalyst has a higher separation rate of photo-generated charges than the 6WO_3_/CN composite and bare CN, which is in accordance to the PL, SPV, FL, and *I*–*V* results.

### Photocatalytic Performance

**Fig. 6:**
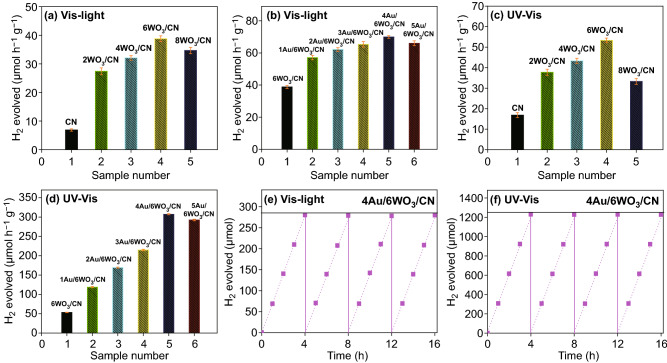
H_2_ production activity of **a** CN and *x*WO_3_/CN photocatalysts under visible light, **b** 6WO_3_/CN and *y*Au/6WO_3_/CN photocatalysts under visible light, **c** CN and *x*WO_3_/CN photocatalysts under UV–visible light, and **d** 6WO_3_/CN and *y*Au/6WO_3_/CN photocatalysts under UV–visible light. Error bars are added to Fig. 6**a**–**d**. Photocatalytic stability and recyclability test of the 4Au/6WO_3_/CN composite for H_2_ production **e** under visible light and **f** under UV–visible light

The catalytic activities of CN, *x*WO_3_/CN and *y*Au/6WO_3_/CN photocatalysts were evaluated for H_2_ production under UV–visible and visible-light illumination. As obvious from Fig. 6a, CN produced a small amount of H_2_ (6.87 μmol h^−1^ g^−1^) under visible-light illumination. It should be noted that the amount of H_2_ produced over *x*WO_3_/CN composites is remarkably enhanced and the 6WO_3_/CN composite produced 38.77 μmol h^−1^ g^−1^ of H_2_. Figure 6b shows that after photo-deposition of Au nanoparticles, the visible-light photoactivity is further improved and the amount optimized 4Au/6WO_3_/CN composite produced 69.9 μmol h^−1^ g^−1^ of H_2_. The quantum efficiencies of the CN, 6WO_3_/CN, and 4Au/6WO_3_/CN photocatalysts for H_2_ evolution at wavelength 420 nm are calculated to be 0.68%, 3.0%, and 4.17%, respectively. The quantum efficiency yield of 4Au/6WO_3_/CN composite is much higher than the reported correlated systems as shown in Table S1. The H_2_ production activities were further evaluated under UV–visible illumination. Figure 6c shows the H_2_ production activities of CN and *x*WO_3_/CN photocatalysts under UV–visible illumination. One can see that CN produced 16.87 μmol h^−1^ g^−1^ of H_2_ under UV–visible light which is much higher than that produced under visible light. The activity of *x*WO_3_/CN composite for H_2_ production under UV–visible illumination is greatly improved and the amount optimized 6WO_3_/CN composite produced 53.32 μmol h^−1^ g^−1^ of H_2_. It must be noted from Fig. 6d that the H_2_ production over *y*Au/6WO_3_/CN composites under UV–visible illumination is much significant and the amount optimized 4Au/6WO_3_/CN is capable of producing 307.36 μmol h^−1^ g^−1^ of H_2_. Thus, the significantly improved photocatalytic activities of the amount optimized 4Au/6WO_3_/CN composite pointing out the favorable synergetic effect of the well-designed three-component photocatalyst. The photocatalytic stability and recyclability tests of the amount optimized 4Au/6WO_3_/CN composite for H_2_ evolution were also carried out under UV–visible and visible-light irradiation. Figure 6e, f indicates that the photocatalyst does not show any obvious decrease in the photocatalytic activity under visible and UV–visible irradiation, even after 4 photocatalytic recycles (each of 4 h). This confirms the high photostability of the designed three components system. To elucidate the change in the crystal structure of 4Au/6WO_3_/CN composite before and after four consecutive photocatalytic cycles of H_2_ evolution under visible-light irradiation, XRD patterns were measured as shown in Fig. S21. As can be seen, there is no obvious change in the crystal structure before and after the photocatalytic reaction. The result specifies excellent recycling performance and stability of the 4Au/6WO_3_/CN composite.

To test the improved photocatalytic performance, 2,4-DCP photodegradation was also carried out under visible-light irradiation. In heterogeneous photocatalysis, the adsorption of pollutants on the semiconductor surfaces also plays a vital role. Thus, the photocatalytic system was kept in dark under stirring for half an hour to reach the adsorption equilibrium and then irradiated under visible light for 2 h. The adsorption of 2,4-DCP over the optimized CN, 6WO_3_/CN, and 4Au/6WO_3_/CN photocatalysts was 2.2%, 7.9%, and 14.5%, respectively, as shown in Fig. S22a. It is clear from Fig. S22b that degradation rate of 2,4-DCP over CN photocatalyst under visible-light irradiation is 26%. However, the degradation rate is remarkably enhanced after coupling WO_3_, and the optimized 6WO_3_/CN composite degraded 71% of the 2,4-DCP in 2 h. It should be noted from Fig. S22c that the degradation rate of 2,4-DCP over *y*Au/6WO_3_/CN photocatalysts is much significant and 4Au/6WO_3_/CN is capable of degrading 100% 2,4-DCP in 2 h. Thus, it is confirmed that the significantly enhanced photoactivities for H_2_ production and 2,4-DCP degradation are ascribed to the remarkably improved charge carriers separation as investigated by the above photophysical and photochemical results.

### Mechanism Discussion

**Fig. 7:**
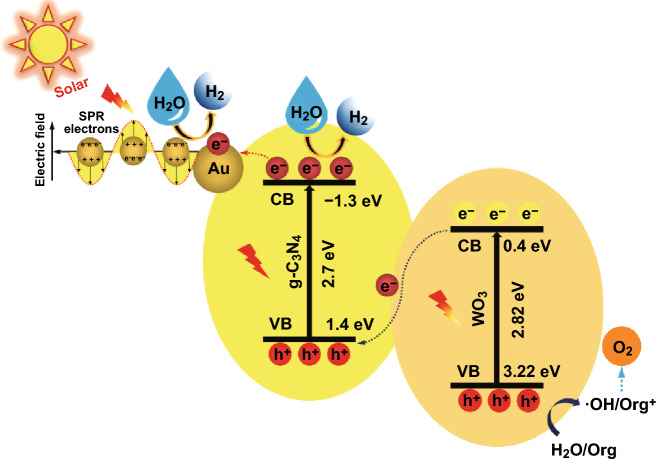
Schematic for the energy band gaps, charge carriers separation and transfer, and the photocatalytic processes over 4Au/6WO_3_/CN composite

According to the above results, a schematic mechanism for charge carriers generation, separation and transfer and the photocatalytic activities over Au deposited Z-scheme WO_3_/CN heterojunction is proposed as depicted in Fig. 7. Accordingly, the conduction-band edges of WO_3_ and CN photocatalysts are located at 0.4 and − 1.3 eV, respectively. Thus, due to the more negative conduction-band potential of CN, its photo-generated electrons can reduce the O_2_ molecules to superoxide radicals (^·^O_2_^−^), while due to the more positive conduction-band potential of WO_3_, its electrons mcan not reduce the O_2_ molecules to ^·^O_2_^−^. The reason is that the standard redox potential of O_2_/^·^O_2_^−^ is − 0.046 eV [18]. In contrast, the valence band edges of WO_3_ and CN photocatalysts are located at 3.22 and 1.4 eV, respectively. Hence, the valence band potential of WO_3_ can oxidize the OH^−^ to ^·^OH as confirmed by the fluorescence results, while that of the CN can not oxide because the standard redox potential of OH^−^/^·^OH is 2.7 eV [18]. To further confirm the ^·^OH formation, the EPR spectroscopy analysis of the optimized 4Au/6WO_3_/CN photocatalyst was carried out in dark and under visible-light irradiation in the presence of trapping reagent DMPO. As shown in Fig. S22d, no characteristic peaks of DMPO-^·^OH can be observed in dark. However, under visible-light irradiation, the characteristic peaks of DMPO-^·^OH can be clearly observed and their intensities steadily increased with the increase in irradiation period, i.e., 5, 10, 15, and 20 min. These results further specify that ^·^OH plays a vital role in the degradation of 2,4-DCP.

Thus, the composite of WO_3_ and CN directs a Z-scheme heterojunction. Based on the above facts, it is concluded that when Au deposited WO_3_/CN Z-scheme heterojunction is fabricated and irradiated under UV–visible and/or visible light, photo-generated charge carriers are produced. Meanwhile, the electrons are excited to the conduction bands while leaving photo-generated-holes in valence bands. Thus, the excited electrons in the conduction band of WO_3_ and the holes left in the valence band of CN quickly recombine at the interface. On the other hand, the photo-excited electrons in the conduction band of CN would transfer to Au and the excited holes left in the valence band of WO_3_ would transfer to its surface, resulting in the drastically improved carrier’s separation. Subsequently, the electrons on the surface of Au would induce water reduction to evolve H_2_ and the photo-generated holes on the surface of WO_3_ would oxidize OH^−^ to generate ^·^OH. The generated ^·^OH will directly oxidize the 2,4-DCP pollutant molecules. The above results reveal that decoration of Au on WO_3_/CN Z-scheme heterojunction can significantly promote charge carriers separation and transfer leading to the drastically promoted photocatalytic performance for water reduction and pollutant oxidation.

In brief, the individual g-C_3_N_4_ can produce a little amount of H_2_ due to its suitable reduction potential for water reduction. However, WO_3_ cannot commence water reduction reactions because its conduction-band potential is more positive than the standard reduction potential value of H_2_O (i.e., 0 eV) versus the normal hydrogen electrode (NHE). In addition, the composite of WO_3_ and CN can remarkably improve the photocatalytic H_2_O reduction to evolve H_2_, especially the Au decorated WO_3_/g-C_3_N_4_ composite. In this case, the VB of WO_3_ causes photo-oxidation of water and CB of g-C_3_N_4_ is responsible for photo-reduction of water and produce H_2_. The experimentally observed and theoretically simulated band edge positions of these materials have strong correlation with each other and their energy levels nicely satisfy the Z-scheme system.

## Conclusions

In summary, Au decorated WO_3_/CN Z-scheme heterojunctions have been successfully fabricated for the first time that exhibited superior photocatalytic performance for solar H_2_ production and 2,4-DCP degradation. The enhanced photocatalytic performance is ascribed to the promoted charge separation by constructing Z-scheme heterojunction and to the SPR effect of Au nanoparticles. The calculated quantum efficiency for H_2_ evolution at a wavelength 420 nm was 4.17%. Finally, our theoretical and experimental data have a nice correlation with each other that further confirms and validates the enhanced photocatalytic performance of 4Au/6WO_3_/CN nanocomposite. This work would be helpful for the future development of CN-based highly proficient catalysts and will be of great significance to meet the energy and environmental demands.

## Electronic supplementary material

Below is the link to the electronic supplementary material.

Supplementary material 1 (PDF 2340 kb)
