# Supplementary material for: Experimental and DFT Studies of Au Deposition Over WO3/g-C3N4 Z-Scheme Heterojunction
Source: Nanomicro Lett. 2019 Dec 19;12:7. doi: 10.1007/s40820-019-0345-2 (PMC7770730; doi:10.1007/s40820-019-0345-2)
Supplement: Supplementary file 1 — Supplementary material 1 (PDF 2340 kb) [file 40820_2019_345_MOESM1_ESM.pdf]

Supporting Information for

Experimental and DFT Studies of Au Deposition Over WO_3/g-C_3N_4 Z-Scheme Heterojunction

Muhammad Humayun[1, 2], Habib Ullah[3], Junhao Cao[1], Wenbo Pi[1], Yang Yuan[1], Sher Ali[1], Asif Ali Tahir[3], Pang Yue[4], Abbas Khan[5], Zhiping Zheng[1], Qiuyun Fu[1], Wei Luo[1, 2, *]

[1]Engineering Research Center for Functional Ceramics of the Ministry of Education, School of Optical and Electronic Information, Huazhong University of Science and Technology, Wuhan 430074, People's Republic of China

[2]China-EU Institute for Clean and Renewable Energy, Huazhong University of Science and Technology, Wuhan 430074, People's Republic of China

[3]Environment and Sustainability Institute (ESI), University of Exeter, Penryn Campus, Penryn, Cornwall TR10 9FE, United Kingdom

[4]College of Engineering and Applied Sciences, Nanjing University, Nanjing, 210093, People's Republic of China

[5]Department of Chemistry, Abdul Wali Khan University, Mardan 23200, Khyber Pakhtunkhwa, Pakistan

*Corresponding author. E-mail: luowei@mail.hust.edu.cn (Wei Luo)

Supplementary Figures

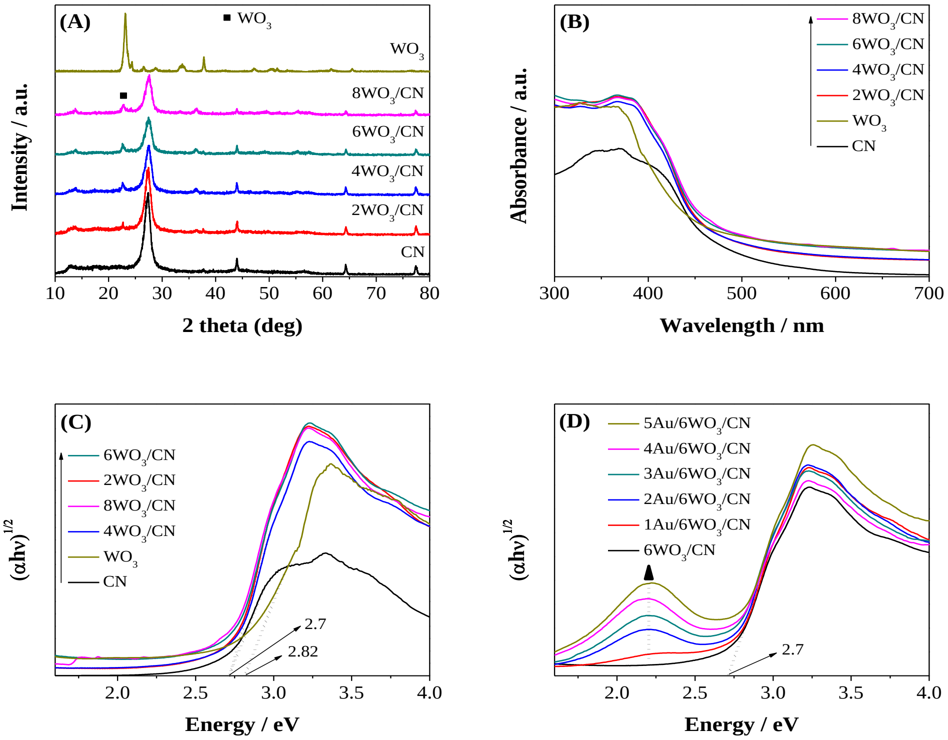

Fig. S1 X-ray diffraction (XRD) patterns (**A**), and UV-Vis absorption spectra (**B**) of CN, WO_3 and xWO_3/CN photocatalysts. The estimated band gaps from the intercept of tangents to the plots of $(\alpha h\nu)^{1/2}$ versus photon energy (hν) of CN, WO_3 and xWO_3/CN photocatalysts (**C**) and of $6WO_3$/CN and $yAu/6WO_3$/CN photocatalysts (**D**)

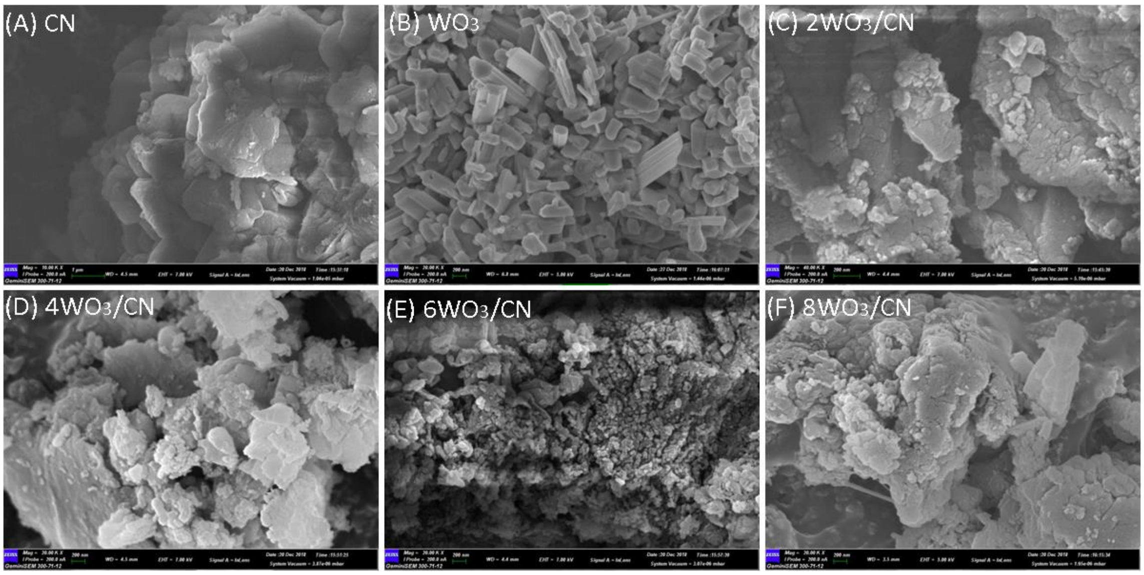

Fig. S2 Scanning electron microscopy (SEM) images (**A**) of CN, (**B**) of WO_3, and (**C**-**F**) of xWO_3/CN composites

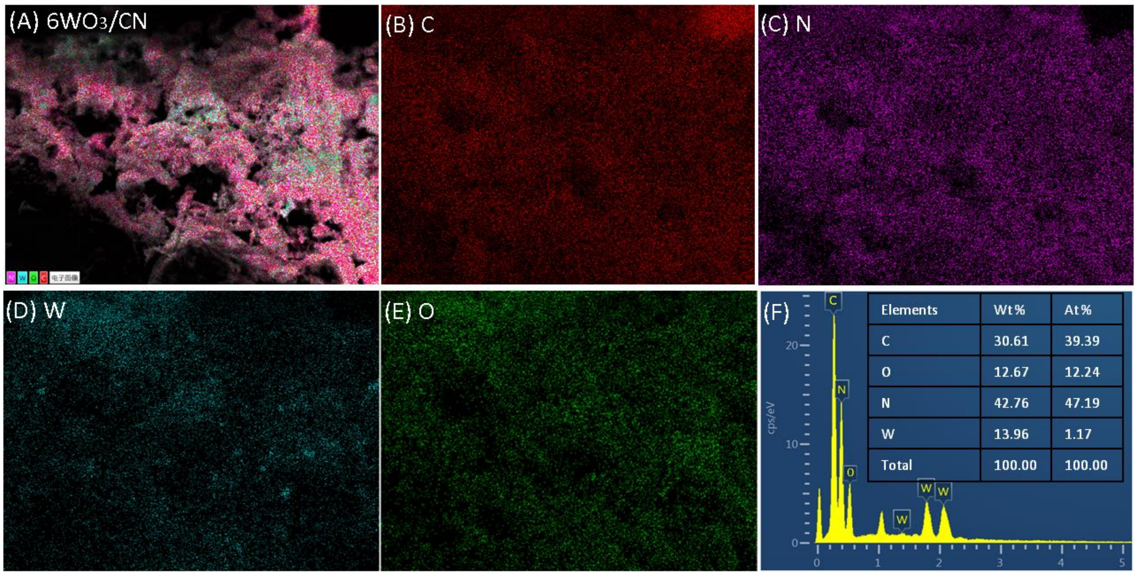

Fig. S3. Energy dispersive X-ray spectroscopy (EDS) elemental mapping images (**A**) of $6WO_3/CN$ composite, (**B**) of C element, (**C**) of N element, (**D**) of W element and (**E**) of O element. Energy dispersive X-ray (EDX) spectrum (F) of $6WO_3/CN$ composite

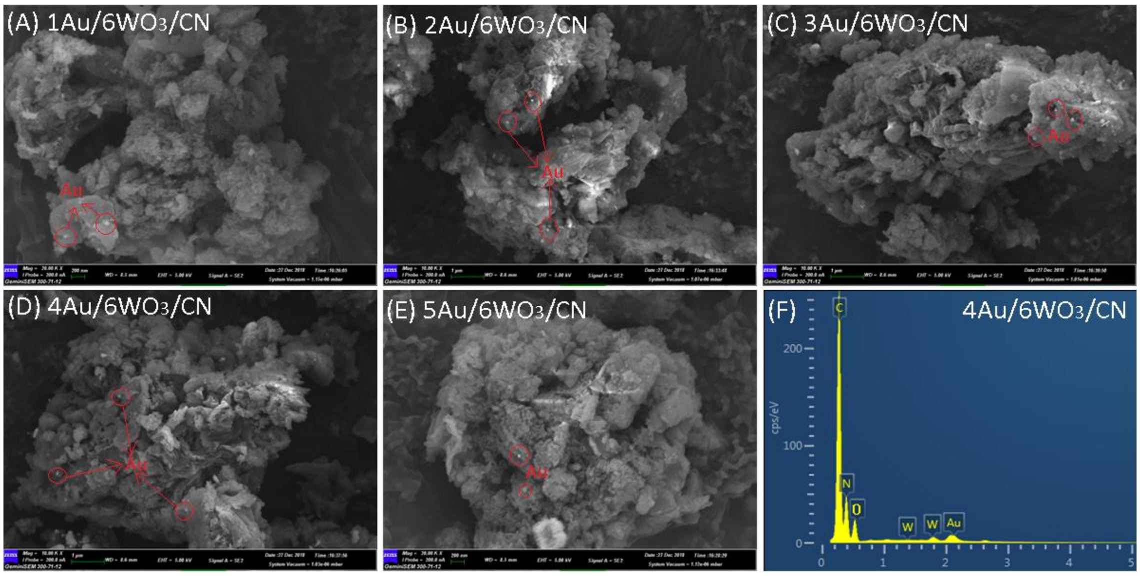

Fig. S4 Scanning electron microscopy (SEM) images (**A-E**) of $yAu/6WO_3/CN$ nanocomposites. Energy dispersive X-ray (EDX) spectrum (**F**) of $4Au/6WO_3/CN$ composite

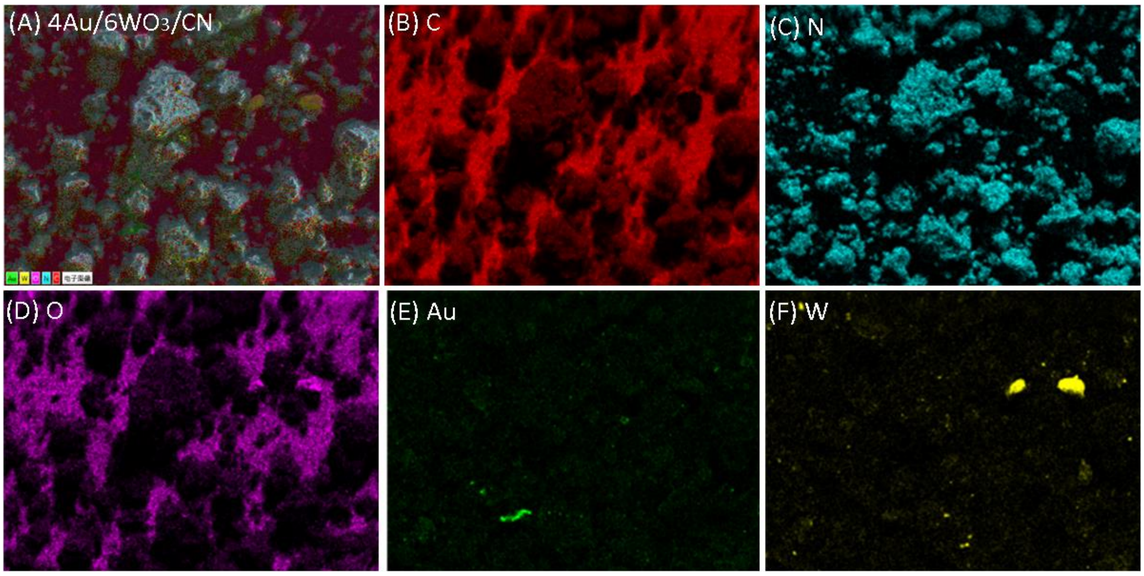

Fig. S5. Energy dispersive X-ray spectroscopy (EDS) elemental mapping images (**A**) of $6WO_3/CN$ composite, (**B**) of C element, (**C**) of N element, (**D**) of O element (**E**) of Au and (**F**) of W element

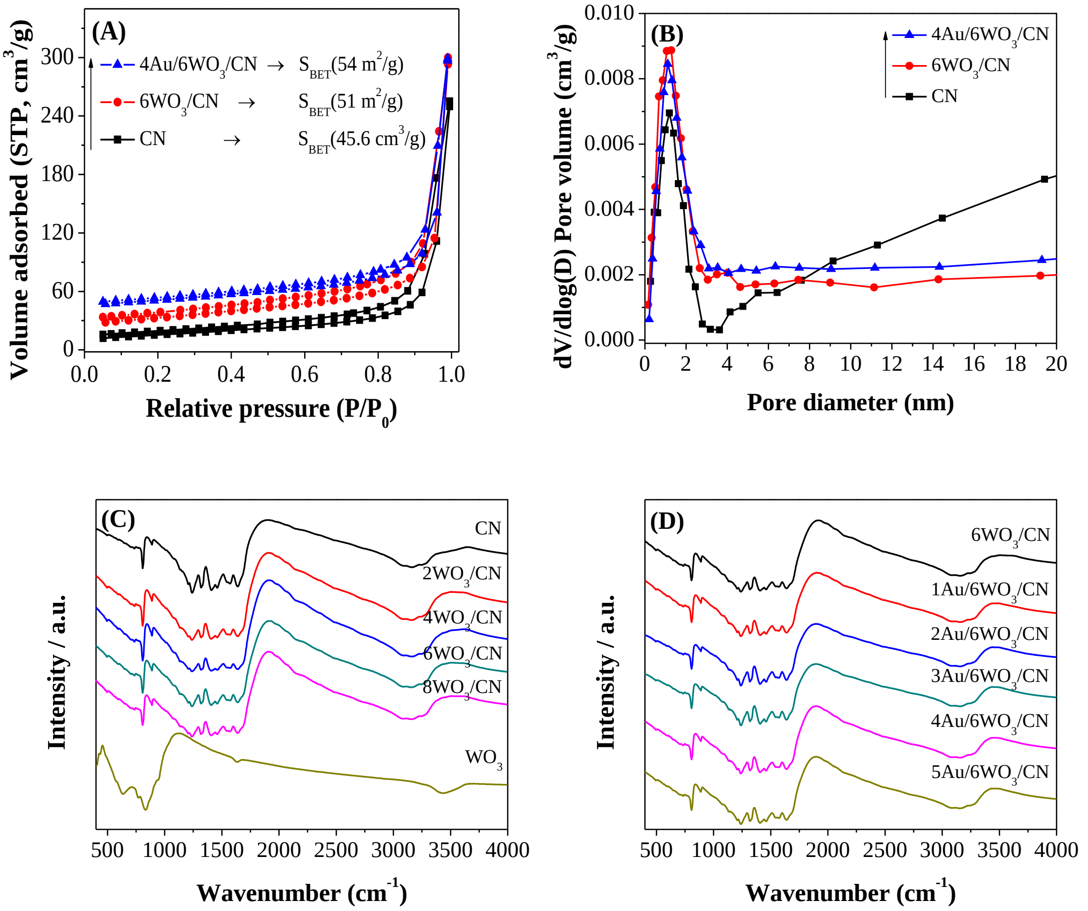

Fig. S6 N_2 adsorption-desorption isotherm curves (**A**) and pore diameter distribution (**B**) of CN, $6WO_3$/CN and $4Au/6WO_3$/CN photocatalysts, FT-IR spectra of CN and xWO_3/CN photocatalysts (**C**) FTIR spectra of $6WO_3$/CN and $yAu/6WO_3$/CN photocatalysts (**D**)

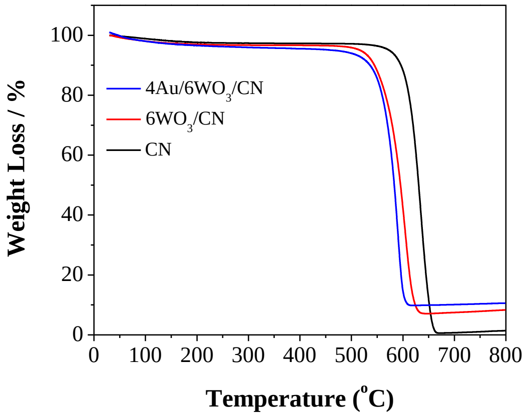

Fig. S7 Thermogravimetric analysis of CN, $6WO_3$/CN and $4Au/6WO_3$/CN photocatalysts under air conditions

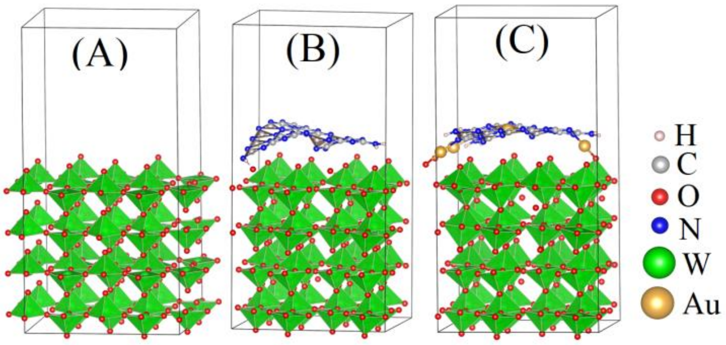

Fig. S8 The Optimized structures (**A**) of WO_3, (**B**) of $6WO_3$/CN and (**C**) of $4Au/6WO_3$/CN

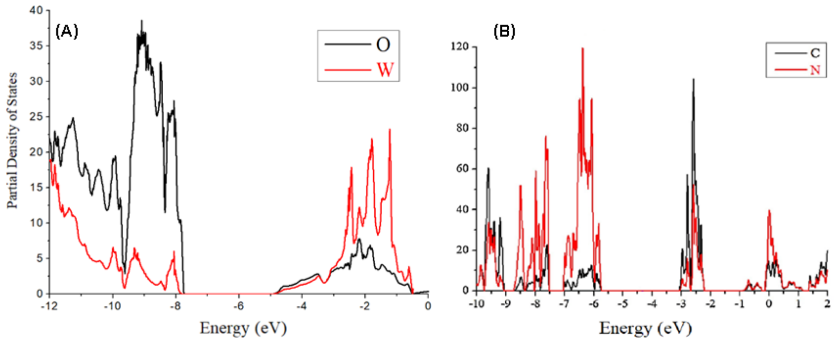

Fig. S9 Partial density of states (**A**) of W and O atoms in WO_3 and (**B**) of C and N atoms in CN; energy values are versus vacuum

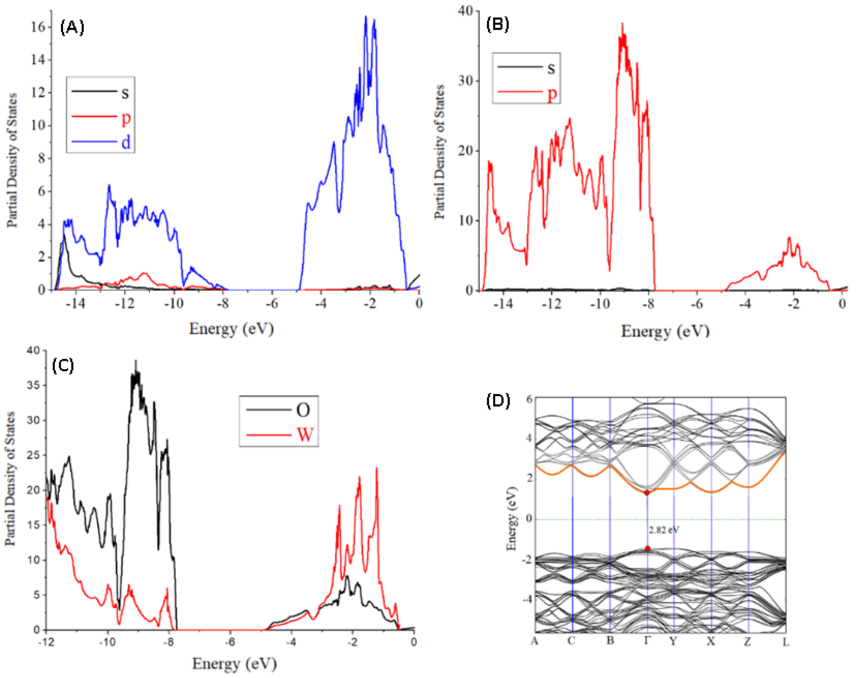

Fig. S10 Partial density of state of s, p orbitals of O (**A**), s, p, d orbitals of W atoms (**B**), W, O atoms (**C**), and band structure of the unit cell of WO_3 (**D**)

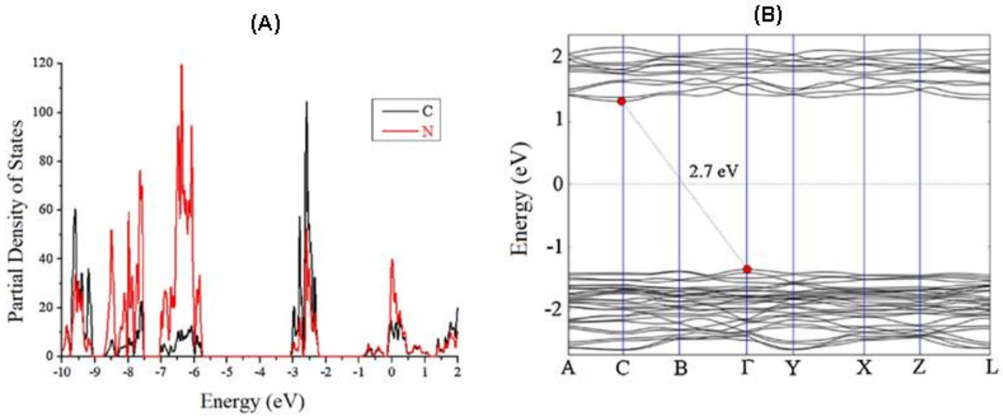

Fig. S11 Partial density of state of C and N (**A**) and the band structure of CN (**B**)

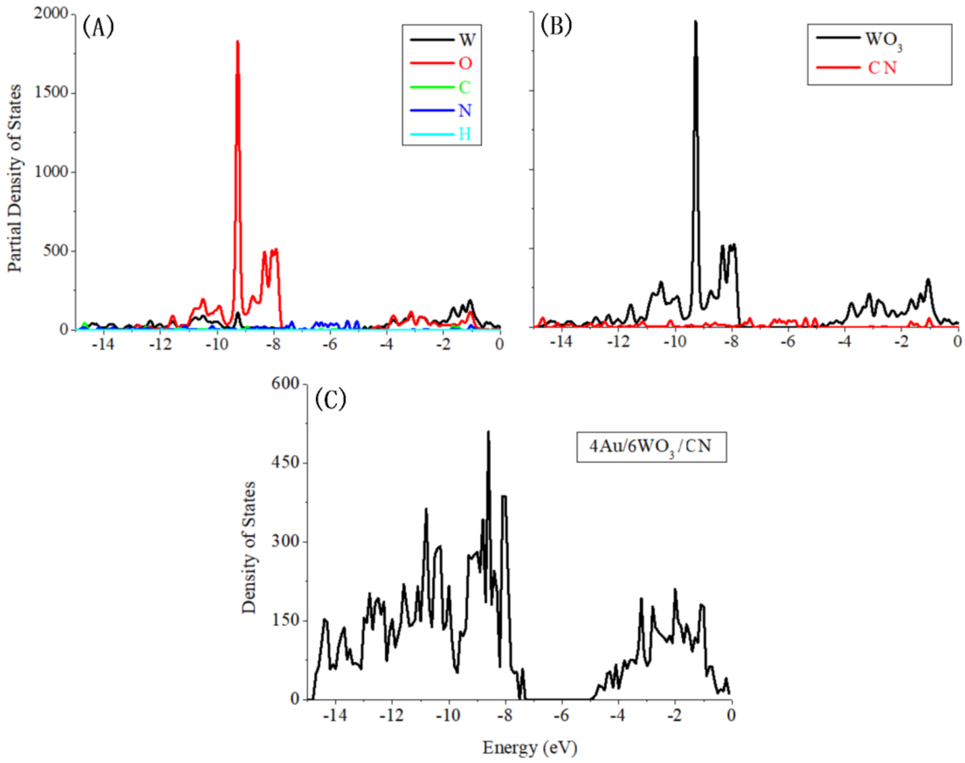

Fig. S12 PDOS (**A**) of W, O, C, N, and H, (**B**) of WO_3 and CN and (**C**) DOS of $6WO_3$/CN catalysts

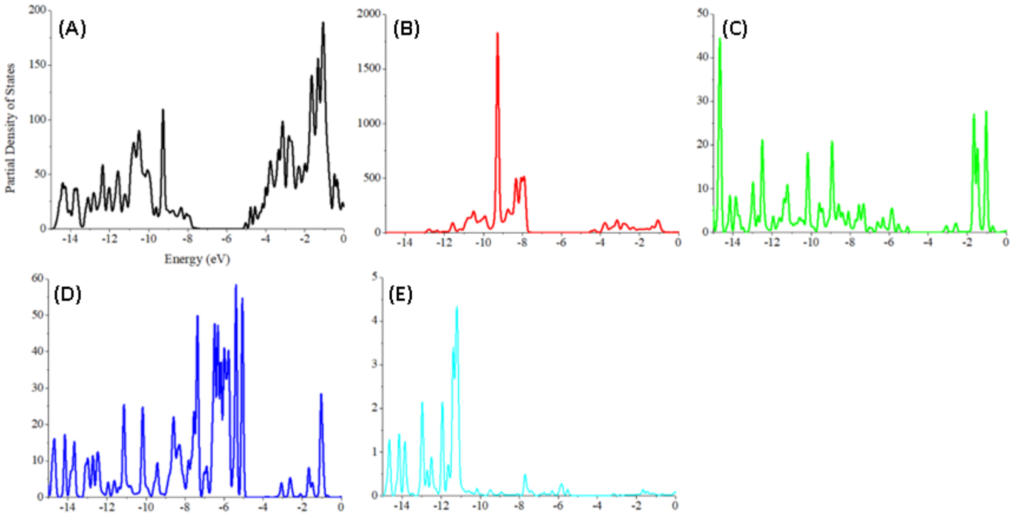

Fig. S13 PDOS of W (**A**), O (**B**), C (**C**), N (**D**), and H (**E**) in $6WO_3$/CN

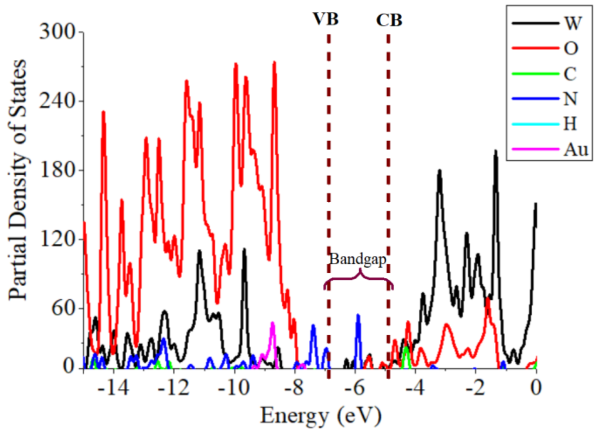

Fig. S14 PDOS of W, O, C, N, H, and Au in $4Au/6WO_3/CN$ composite

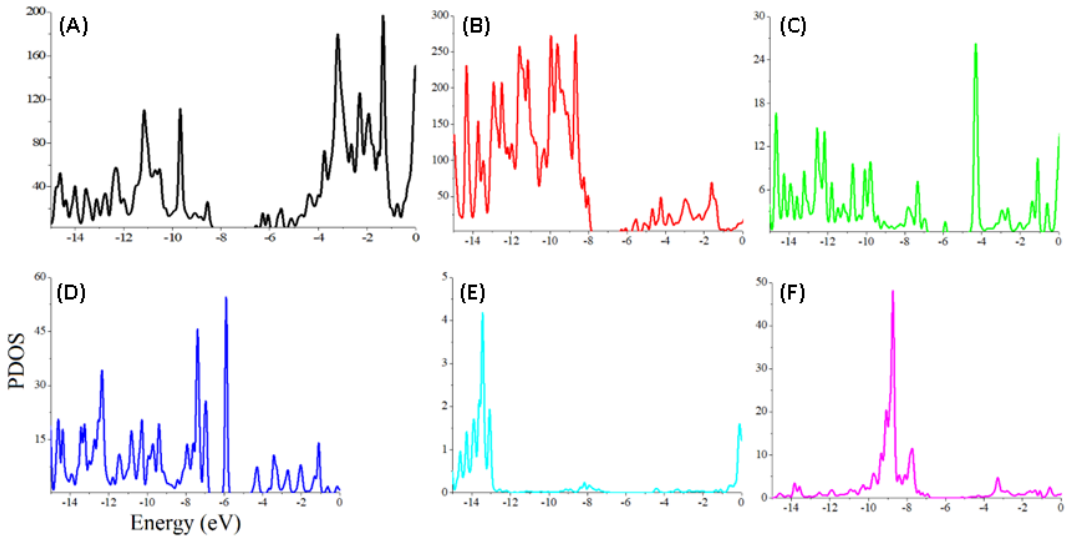

Fig. S15 PDOS of W (**A**), O (**B**), C (**C**), N (**D**), H (**E**), and Au (**F**) in $4Au/6WO_3/CN$

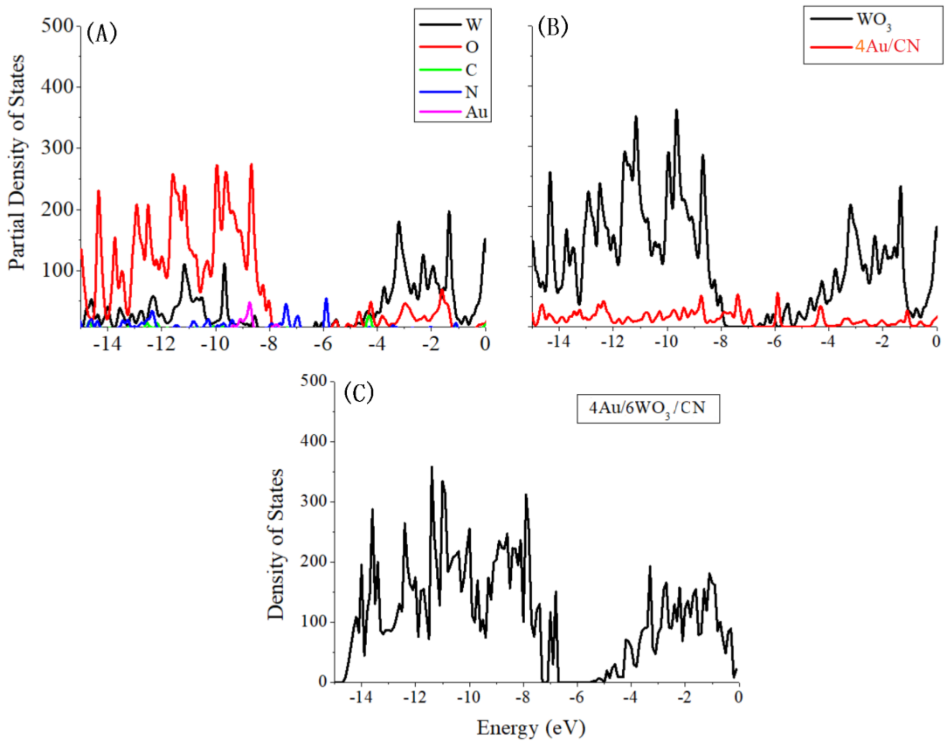

Fig. S16 PDOS of W, O, C, N, H, and Au (**A**), WO_3 and Au/CN (**B**) and DOS of 4Au/6WO_3/CN (**C**)

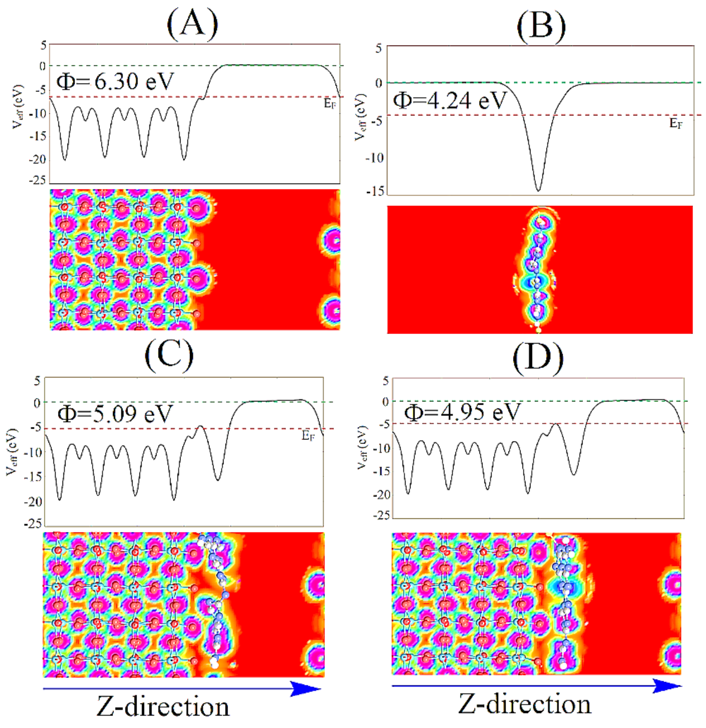

Fig. S17 Electrostatic potentials map (**A**) of WO_3, (**B**) of Au/CN, (**C**) of 6WO_3/CN and (**D**) of 4Au/6WO_3/CN photocatalysts. The red dashed lines denote Fermi energy level and green represents vacuum energy level

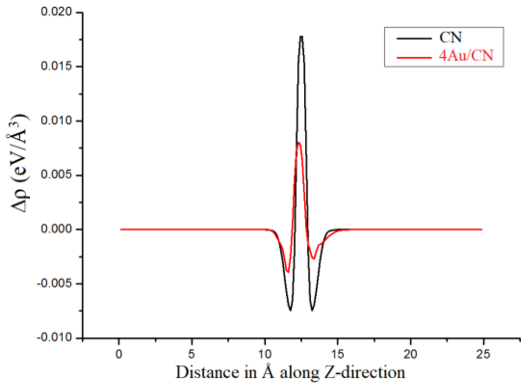

Fig. S18 Electron difference density plots of CN and Au/CN

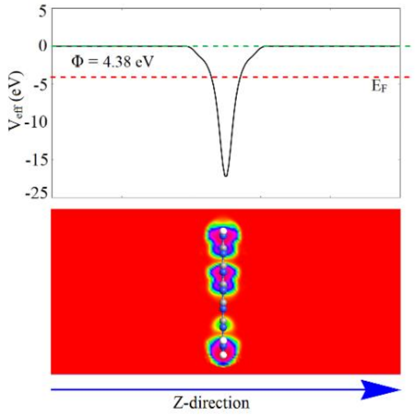

Fig. S19 Electrostatic potential map of CN photocatalyst along Z-direction

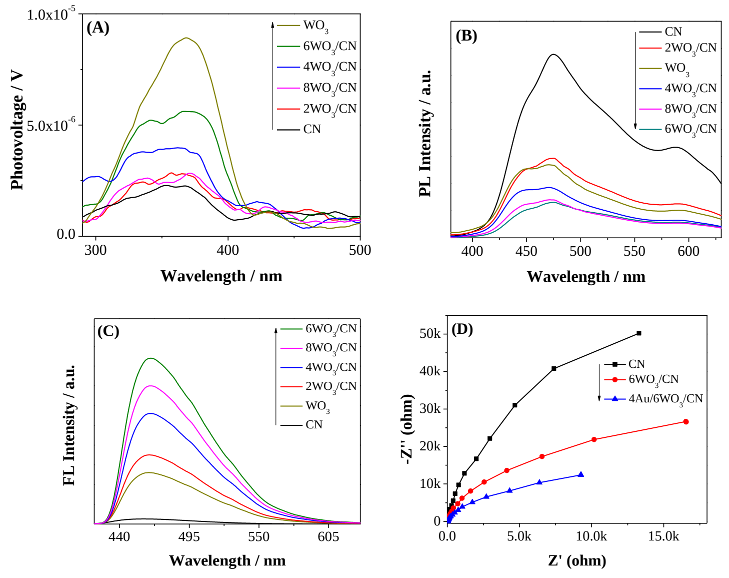

Fig. S20 Surface photovoltage (SPV) spectra (**A**), photoluminescence (PL) spectra (**B**) and fluorescence (FL) spectra related to the •OH amount (**C**) of CN, WO_3 and

xWO_3/CN composites. Electrochemical impedance spectroscopy (EIS) spectra (**D**) of CN, $6WO_3$/CN, and $4Au/6WO_3$/CN samples under visible-light irradiation

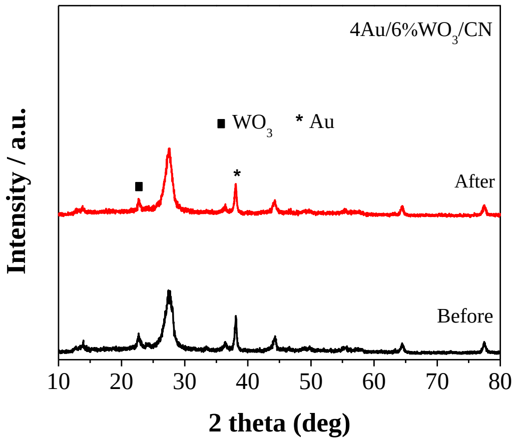

Fig. S21 XRD patterns of the $4Au/6WO_3$/CN composite before and after four consecutive photocatalytic cycles of H2 evolution under visible-light irradiation

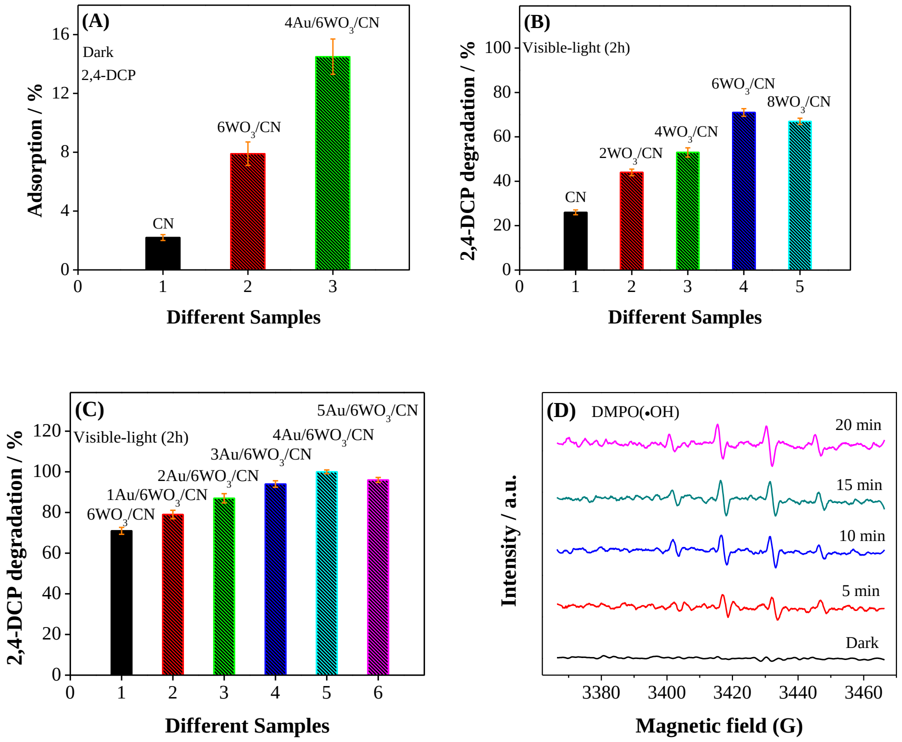

Fig. S22 Adsorption of 2,4-DCP over CN, $6WO_3$/CN, and $4Au/6WO_3$/CN photocatalysts (**A**), Visible-light catalytic activities for 2,4-DCP degradation (**B**) of CN and xWO_3/CN composites and (**C**) of $6WO_3$/CN and $yAu/6WO_3$/CN composites. Error bars are added to Fig. S22 (**A-C**). EPR spectra of hydroxyl radical (•OH) adduct trapped by DMPO in the presence of $4Au/6WO_3$/CN photocatalyst in dark and under visible light irradiation (**D**)

Table S1 Comparison of our H_2 production results with the previous reports

S.#	Photocatalysts	Source of light	Wavelength used	Quantum efficiency (%)	References
1	Pt/CoTiO3/g-C3N4	300 Xe-lamp	420 nm	3.23%	ACS Appl. Mater. Interfaces, 2016, 8, 13879–13889
2	Au/g-C3N4/NiFe2O4	300 Xe-lamp	420 nm	1.12%	RSC Adv. 2016, 6, 54964–54975
3	1 Au-6T/6S-PCN	300 Xe-lamp	420 nm	3.34%	Appl. Catal., B 2018, 237, 1082–1090
4	0.2 % MoS2/g-C3N4	300 Xe-lamp	420 nm	2.1%	Angew. Chem., Int. Ed. 2013, 52, 3621−3625
5	NiS/g-C3N4	300 Xe-lamp	440 nm	1.9%	ChemSusChem. 2013, 6, 2263–2268
6	GS/g-C3N4	300 Xe-lamp	λ>420	2.6%	J. Phys. Chem. C 2011, 115, 7355–7363
7	4Au/6WO3/CN	300 Xe-lamp	420 nm	4.17%	Current Work

S2 Quantum Efficiency Calculations for H_2 Production

The calculated quantum efficiencies of CN, $6WO_3$/CN and $4Au/6WO_3$/CN photocatalysts for H_2 evolution at wavelength 420 nm are given below. The light source was a 300 W Xe-lamp and the photocatalysts were irradiated under 420 nm wavelength for 8 hours. The average incident irradiation was determined to be 2.01 mW/cm^2 by Newport (Oriel instrument USA-model-91150V ser. No 391/0118) and the area of light collector part was 6.5 cm^2. The H_2 amount produced over CN, $6WO_3$/CN and $4Au/6WO_3$/CN photocatalysts under wavelength 420 nm for 8 hours was 4.5, 19.7, and 27.4 μmol, respectively.

Quantum efficiency calculation for CN, $6WO_3$/CN and $4Au/6WO_3$/CN photocatalysts at λ=420 nm: Number of incident photons (N) in 8 h over 6.5 cm^2 area:

$$N = \frac{E\lambda}{hc} = \frac{2.01 \times 10^{-3} \times 6.5 \times 420 \times 10^{-9} \times 8 \times 3600}{6.626 \times 10^{-34} \times 3 \times 10^{8}} = 7.9 \times 10^{20}$$

$$QE = \frac{2 \times \mu mol\ of\ H_2\ produced \times Avogadro\ number}{the\ number\ of\ incident\ photons} \times 100\%$$

$$QE_{CN} = \frac{2 \times 4.5 \times 10^{-6} \ \times \ 6.02 \times 10^{23}}{7.9 \times 10^{20}} \times 100\% = 0.68\%$$

$$QE_{6WO3/CN} = \frac{2 \times 19.7 \times 10^{-6} \ \times \ 6.02 \times 10^{23}}{7.9 \times 10^{20}} \times 100\% = 3.0\%$$

$$QE_{4Au/6WO3/CN} = \frac{2 \times 27.4 \times 10^{-6} \ \times \ 6.02 \times 10^{23}}{7.9 \times 10^{20}} \times 100\% = 4.17\%$$
